# *S*-HPABA, a marine fungal-derived bioactive compound, exhibits potent lipid-lowering efficacy and safety in modulating lipid dysregulation revealed by integrative lipidomics and molecular biology approaches

**DOI:** 10.3389/fnut.2026.1855150

**Published:** 2026-10-05

**Authors:** Xinming Jia, Menglu Yu, Dongang Shen, Hongyang Fu, Yuqing Wang, Xiao Wang, Jiaoyan Huang, Shilin Sun, Zhiguo Yu, Yuping Chen, Yunli Zhao

**Affiliations:** 1 Department of Pharmaceutical Analysis, School of Pharmacy, Shenyang Pharmaceutical University, Shenyang, China; 2 Key Laboratory of Clinical Research on Respiratory and Digestive Diseases, Baoding Hospital of Beijing Children’s Hospital Affiliated to Capital Medical University, Baoding, Hebei, China

**Keywords:** functional food, lipid-lowering effect, lipidomics, marine fungus, multitargets mechanism, *S*-2-(2-hydroxypropanamido) benzoic acid

## Abstract

**Introduction:**

Hyperlipidemia is a widespread metabolic disorder that places a heavy burden on normal body functions. Given the drawbacks of current lipid-lowering drugs, there is considerable interest in developing alternative nutraceuticals from natural sources. *S*-2-(2-hydroxypropanamido) benzoic acid (*S*-HPABA) is a novel compound obtained for the first time from a marine fungus, which has demonstrated strong lipid-lowering effects *in vitro* and *in vivo* with an excellent safety profile.

**Methods:**

To investigate *S*-HPABA’s mechanism of action, an integrated strategy of lipidomics and bioinformatics was adopted to screen candidate targets. The selected targets were then validated through RT-qPCR and western blotting.

**Results:**

The results indicated that 57 differential metabolites and 11 metabolic pathways were significantly regulated by *S*-HPABA. Based on the combined analyses, five targets were initially selected. Validation experiments showed that FASN, PLA2G2A and TYMP play crucial roles in the lipid-lowering process, with FASN being the most prominent.

**Discussion:**

Mechanistically, *S*-HPABA-induced reduction of lipid accumulation was associated with decreased FASN expression and PI3K/AKT/mTOR pathway inhibition. In summary, *S*-HPABA is a safe and potent lipid-lowering bioactive compound with promise as a functional food ingredient for metabolic health.

## Graphical abstract

**Figure:**
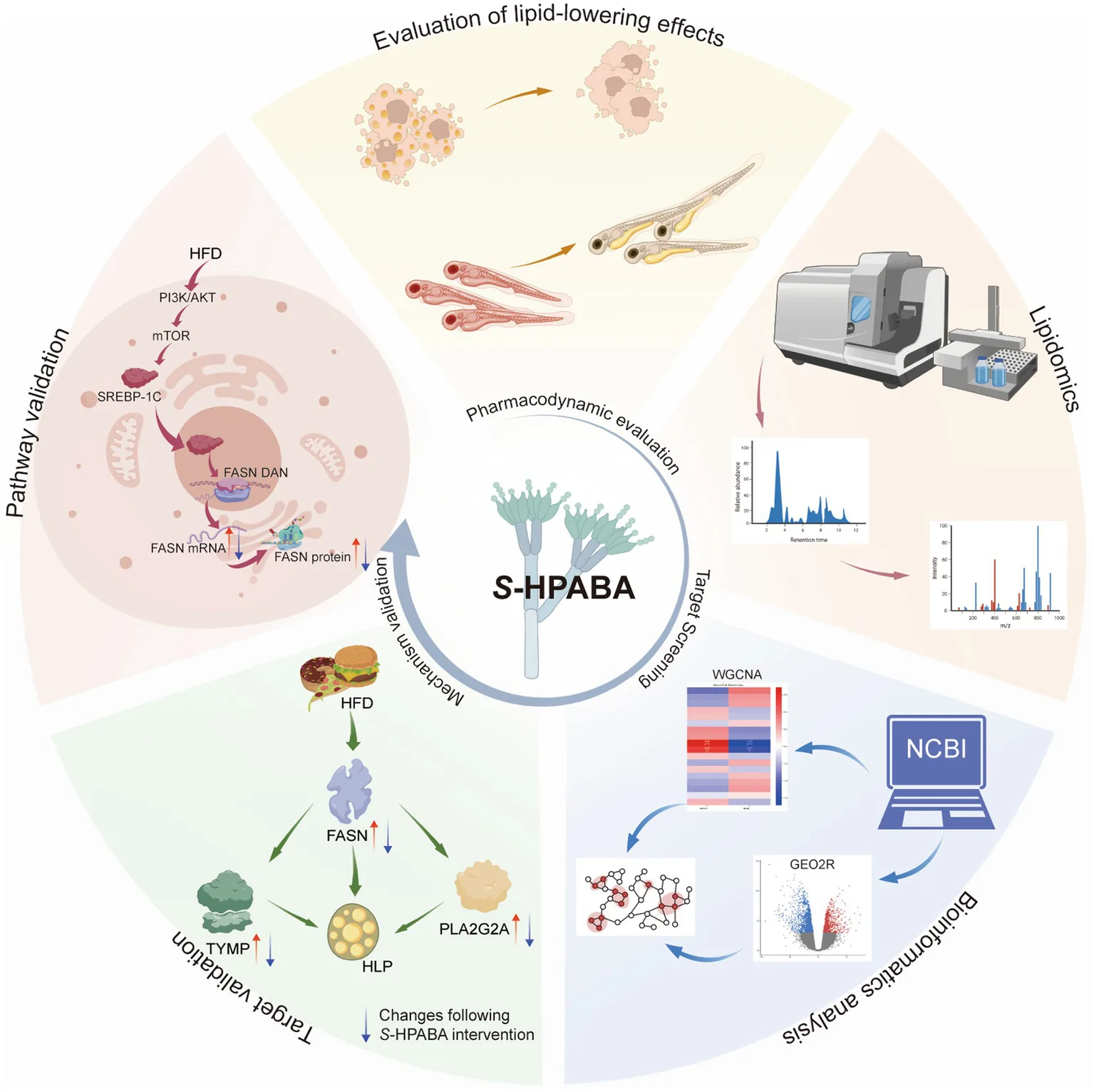

## 1 Introduction

Hyperlipidemia (HLP), a common chronic metabolic disorder, is caused by a disruption in the balance of fat intake and utilization (1, 2). As a potent risk factor, HLP markedly increases the susceptibility to comorbidities, including atherosclerosis, fatty liver and diabetes, ultimately progressing to serious metabolic diseases and cardiocerebrovascular diseases (3–5). For instance, numerous clinical trials have demonstrated that HLP is a major risk factor for myocardial infarction and heart failure (6, 7). Meanwhile, previous studies have shown that HLP is a primary triggering factor for non-alcoholic fatty liver disease, and reducing lipid levels could effectively prevent its onset (8). In recent years, the escalating global prevalence of HLP has imposed a dual burden: deteriorating patient quality of life and mounting pressure on healthcare systems.

At present, there are two mainstream treatment strategies for HLP, namely lifestyle interventions and pharmacotherapy (9, 10). The former is safe and low-cost, but its effects are slow to manifest, often poorly sustained, and subject to considerable interindividual variability. The latter offers rapid lipid-lowering effects but carries side effects. Consequently, the combined approach of long-term lifestyle management and precise drug treatment has been widely adopted. Currently, statins, fibrates, ezetimibe, bile acid sequestrants and niacin are commonly used lipid-lowering agents (11, 12). Among them, statins are the only class included in the World Health Organization’s list of essential medicines (13). However, they have serious hepatotoxicity, muscle toxicity, gastrointestinal adverse reactions, and drug interactions (14–17). Among the adverse symptoms of non-statin drugs, gastrointestinal side effects are the most frequently reported (18). Collectively, there is a pressing need to develop novel lipid-lowering functional food ingredients or bioactive compounds that combine excellent efficacy with favorable safety profiles.

The rapid advancement of technology has transformed rich marine resources into a treasure trove for the discovery of new bioactive compounds with metabolic benefits, offering unprecedented opportunities for improving human health. The extreme diversity of the marine environment drives the evolution of unique metabolic pathways, enabling organisms to generate a vast array of novel bioactive compounds (19, 20). These natural products possess a wide spectrum of pharmacological properties, including antitumor, anti-inflammatory, and metabolic regulatory activities (21–23). Notably, a variety of marine-derived bioactive compounds have been successfully translated into applications, highlighting the immense potential of marine resources in addressing metabolic health challenges (24–28). It is anticipated that marine resources will not only provide bioactive compounds but also inspire the design and optimization of functional food ingredients. Therefore, the exploration of marine-derived compounds represents a promising direction, which would contribute to the innovation and diversification of strategies for managing lipid disorders in the future.

A novel ingredient, designated *S*-2-(2-hydroxypropanamido) benzoic acid (*S*-HPABA), was isolated and characterized from the metabolites of *Penicillium chrysogenum* derived from a marine environment. Early studies revealed that *S*-HPABA exhibited a broad spectrum of activities, including anti-inflammatory, antiplatelet and antithrombotic effects (29, 30). Our previous research has established both the biosynthetic and chemical synthesis of *S*-HPABA (31). Among these, the second approach was the most commonly used (Supplementary Figure S1). In the latest experiment, *S*-HPABA exhibited remarkable lipid-lowering efficacy alongside minimal cytotoxicity, which gives it a bright future for nutritional or nutraceutical applications. To elucidate its mechanism of action, we established an approach combining lipidomics experiments with database information analysis. This study provides comprehensive evidence supporting the lipid-lowering effects of *S*-HPABA, thereby laying a solid foundation for its future development as a functional food ingredient or dietary supplement for managing lipid disorders.

## 2 Materials and methods

### 2.1 Materials

*S*-HPABA (≥ 98%) was prepared in our laboratory, and simvastatin (Sim, ≥ 98%) was purchased from Macklin (Shanghai, China). Oil red O was produced by Solarbio (Beijing, China). The CCK-8, TG and TC kits belong to the Nanjing JianCheng (Nanjing, China). The sodium oleate solution was acquired from Siduorui (Shanghai, China). The total RNA extraction kit, reverse transcription kit, and SYBR qPCR Master Mix were purchased from Servicebio (Wuhan, China). The BCA protein assay kit and enhanced chemiluminescence substrate were obtained from Saiwen (Beijing, China). The primary antibodies were provided by UpingBio (Shenzhen, China), Epizyme (Shanghai, China), Proteintech (Wuhan, China) and Cohesion (London, UK).

### 2.2 Cell culture and treatment

#### 2.2.1 HepG2 cell viability, oil red O staining and TC, TG determination

HepG2 cells were seeded and cultured in 96-well plates overnight, and then treated with different concentrations of simvastatin or *S*-HPABA. The cell proliferation rate was assessed by the CCK-8 method after incubating for 24 h.

HepG2 cells were placed into 24-well plates, and followed by being divided into 6 groups, which were the control, model, simvastatin, and *S*-HPABA low, medium, and high dose group. After 12 h, the control group continued to be grown in DMEM, while the remaining five groups were incubated in fresh medium containing 400 μM sodium oleate. Concurrently, simvastatin and different concentrations of *S*-HPABA were added to the administration groups. Oil red O staining was performed after 24 h following the kit steps. In order to calculate the lipid content, each well was covered with 300 μL of isopropanol and incubated for 30 min at 37 °C, and then 150 μL was taken from per well to measure the absorbance at 510 nm (32, 33). For TG and TC determining, HepG2 cells were inoculated in 6-well plates, and then exposure treatment was conducted according to the procedure described above. All of the above tests were repeated three times.

#### 2.2.2 3T3-L1 cell viability, oil red O staining and TC, TG determination

3T3-L1 cells were inoculated in 96-well plates at 5 × 10^3^ cells/well, the cytotoxicity of simvastatin and *S*-HPABA were determined with the help of CCK-8 kit.

The 3T3-L1 lipid-loaded cell model was constructed using dexamethasone (Dex), insulin (INS) and 3-isobutyl-1-methylxanthine (IBMX) (34, 35). 3T3-L1 cells were transferred to 24-well plates and divided into 6 groups for oil red O staining. The high-fat model was established except for the control group. Simultaneously, simvastatin and *S*-HPABA were added to the treatment group. Eventually, the stained oil droplet was extracted with isopropanol and the lipid content was measure at 510 nm. In order to analyze the TG and TC contents, 3T3-L1 cells were inoculated in 6-well plates (1 × 10^5^ cells/well) and treated as described above. Both toxicity testing and efficacy evaluation were conducted in triplicate.

### 2.3 Zebrafish culture and testing

#### 2.3.1 Survival rate assessment in zebrafish

Zebrafish (Wild-type AB strain) embryos at 6 h post-fertilization were used for toxicity testing. Embryos (ten per well) were exposed to *S*-HPABA at concentrations of 0, 5, 50, 250, 500, 750 and 1,000 μM. Mortality in each well was recorded at 96 h to calculate the maximum tolerated concentration. Each concentration was tested three times. Subsequently, ten samples were randomly selected from each group, and their heartbeats were counted over a 20-s interval to calculate the heart rate.

#### 2.3.2 Oil red O staining of zebrafish

Zebrafish at 5dpf were divided into six groups for oil red O staining: control, model, simvastatin (1 μM), and *S*-HPABA-treated groups at 10, 50 and 250 μM. Three replicates were included for each group. The zebrafish were fed with E3 solution, and 2% egg yolk was added to the normal rearing except for the control group (36). Simultaneously, simvastatin and *S*-HPABA were blended into the corresponding groups. Upon treatment to 9dpf, six zebrafish which were randomly selected from each group were stained with oil red O and photographed. To calculate the amount of lipid droplet deposition, the images were processed with ImageJ to determine the integrated optical density (IOD).

#### 2.3.3 Evaluation of TC and TG

To determine the effect of *S*-HPABA on TG and TC content in zebrafish, zebrafish at 5dpf were placed in 90 mm culture dishes, and the model was established as described above. After treatment until 9dpf, samples were collected for measurement of TG and TC levels.

### 2.4 Lipidomics in zebrafish

#### 2.4.1 Preparation of samples

The zebrafish samples were homogenized in ice bath with pre-cooled methanol. After the lipid components of the samples were extracted by methyl tert-butyl ether, they were allowed to stand for 30 min and centrifuged for 10 min, and then the supernatant was collected for subsequent testing. The supernatant, which was dried by nitrogen, was redissolved with a mixture of isopropanol and acetonitrile, followed by vortexing and centrifuging. The supernatant was taken for further lipidomics study, while 10 μL was taken in each sample for preparation of quality control (QC) samples.

#### 2.4.2 Instrument conditions and parameters

In the experiment, vanquish UHPLC equipped with a Kinetex EVO-C18 column (100 mm × 2.1 mm, 1.7 μm) was used for chromatographic separations. The heated electric spray ionization (HESI) source was employed, and the mass scanning range was set at m/z 100–1,500. The mobile phase was consisted of acetonitrile: water (9:1, *v*: *v*) solution (A) and acetonitrile: water: isopropanol (1:1:8, *v*: *v*: *v*) solution (B), which contained 0.1% formic acid and 10 mM ammonium formate. The flow rate and column temperature were 0.3 mL·min^−1^ and 40 °C, respectively. After that, 5 μL of each sample was injected into UPLC-Q-Exactive Orbitrap MS (Thermo Fisher, USA) for analysis.

#### 2.4.3 Data processing and analysis

In this study, the raw data was processed with Compound Discoverer software (Thermo Fisher, USA), and the acquired dataset was subsequently imported into SIMCA 14.0 (Umedic, Uppsala, Sweden) for multivariate statistical. Among them, principal component analysis (PCA) and orthogonal partial least squares discrimination analysis (OPLS-DA) were the most commonly used, and the variable importance in projection (VIP) values could be calculated by OPLS-DA. In addition, fold change (FC) and *p* values of intergroup metabolites were obtained through MetaboAnalyst 6.0. Metabolites, which accorded with the criteria of VIP > 1, FC > 1.2 or < 0.8 and *p* < 0.05, were selected as differential lipid components. Subsequently, the differential lipid metabolites were annotated by matching their MS/MS spectra against public databases, including mzCloud, MassBank and Lipid Maps, with a mass tolerance of ± 5 ppm. Finally, pathway enrichment was carried out by inputting differential components into MetaboAnalyst 6.0.

### 2.5 The target screening strategy integrating lipidomics and bioinformatics

The differential metabolites closely associated with metabolic pathways were introduced into Cytoscape. Utilizing the Metascape plugin, a “metabolite-reaction-enzyme-gene” network was constructed to obtain genes linked to these metabolites. The GSE88837 dataset was analyzed with GEO2R and Weighted Gene Co-expression Network Analysis (WGCNA) to obtain obesity-associated genes. Candidate targets which *S*-HPABA may exert its effects were identified by intersecting the genes derived from lipidomics and bioinformatics analyses.

### 2.6 Real-time quantitative PCR (RT-qPCR)

In this section, total RNA isolation was performed using TRIzol reagent alongside a commercial kit optimized for zebrafish samples. After quantification, the RNA was reverse-transcribed into cDNA. Following the reverse transcription, quantitative PCR was carried out on a QuantStudio 1 Plus instrument with GAPDH as the internal control. All mRNA expression levels were normalized to GAPDH, and the 2^−ΔΔCt^ method was used for data analysis. The test was repeated three times for statistical analysis, and the primer sequences are listed in Supplementary Table S1.

### 2.7 Western blot analysis

HepG2 cells were lysed in RIPA buffer containing protease and phosphatase inhibitors, followed by centrifugation. The supernatant was collected, and the protein concentration was determined using the BCA assay. 20 μg of protein per lane were separated by SDS-PAGE and transferred to PVDF membranes. The membranes were blocked with 5% skim milk at room temperature for 2 h, and then incubated with primary antibodies at 4 °C overnight. Thereafter, the membranes were incubated with HRP-conjugated secondary antibodies at 25 °C for 1.5 h. Finally, protein bands were visualized using an ECL kit and quantitatively analyzed with ImageJ software. All experiments were performed in triplicate for each target protein.

The following primary antibodies were used in this section: anti-FASN (R013880), anti-PLA2G2A (CMA4861), anti-TYMP (CQA2296), anti-PI3K (YP-mAb-17844), anti-AKT (CQA8001), anti-mTOR (M900010), anti-phospho-mTOR (R010732), anti-GAPDH (60004-1-Ig), and anti-β-actin (66009-1-Ig).

### 2.8 Statistical analysis

All experiments were performed with at least three biological replicates; the exact numbers are provided in the figure legends. In RT-qPCR experiments, mRNA expression levels were normalized to GAPDH. In western blot analyses, target protein band intensities were normalized to an internal control (GAPDH or β-actin) on the same membrane. All data are presented as mean ± SD. Statistical analysis was performed using GraphPad Prism 10 (San Diego, CA, USA). Significance was assessed by analysis of variance (ANOVA), with *p* < 0.05 considered statistically significant.

## 3 Results

### 3.1 *S*-HPABA sensibly reduced lipid content in HepG2 and 3T3-L1 high-fat cell models

In this study, CCK-8 assay was employed to assess the cytotoxicity of *S*-HPABA and to determine its appropriate administration concentration. As shown in Figures 1A,F, S-HPABA demonstrates a superior safety profile compared to simvastatin in both HepG2 and 3T3-L1 cell lines. Specifically, *S*-HPABA concentrations below 1,250 μM sustained over 80% viability in HepG2 cells, while in 3T3-L1 cells, viability remained above 80% at concentrations as high as 1750 μM.

**Figure 1:**
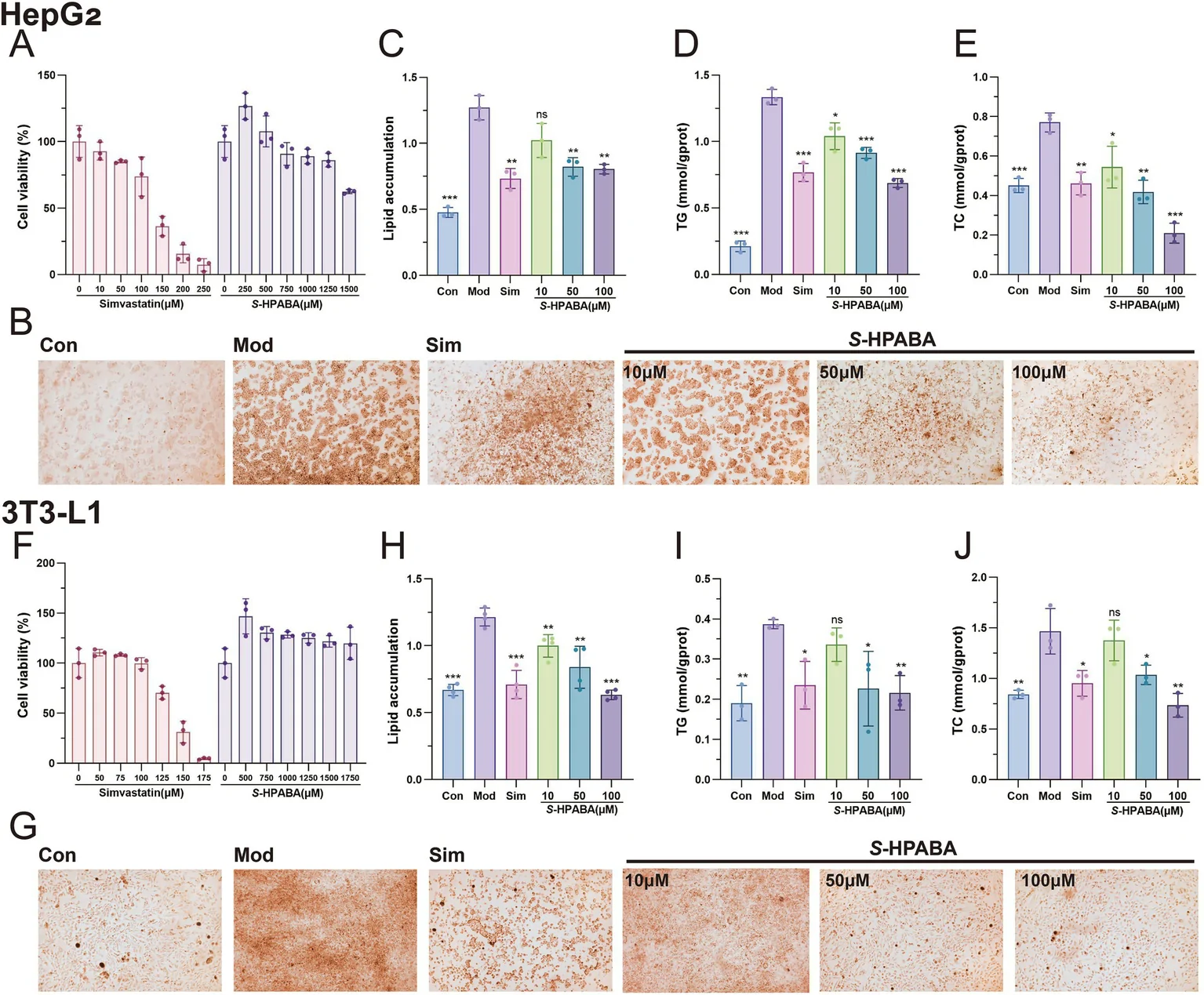
The toxicity and lipid-lowering effects of *S*-HPABA on HepG2 and 3T3-L1 cells. **(A)** Cell viability of *S*-HBAPA on HepG2 cells. **(B)** Oil red O staining of HepG2 cells after *S*-HPABA treatment. **(C)** Quantification of lipid accumulation in HepG2 cells after oil red O staining. **(D,E)** TG and TC levels in HepG2 cells following *S*-HPABA intervention. **(F)** Influence of *S*-HBAPA on 3T3-L1 cell proliferation. **(G)** Oil red O staining and imaging of 3T3-L1 cells. **(H)** Quantify the results of oil red O staining in 3T3-L1 cells. **(I,J)** TG and TC contents of 3T3-L1 cells. The data are presented as mean ± SD (*n* = 3). *p < 0.05, **p < 0.01, ***p < 0.001 vs. the model group.

In pharmacodynamic evaluations, the cytotoxicity of both *S*-HPABA and simvastatin was assessed to ensure that the selected concentrations were non-toxic to cells and to allow a direct efficacy comparison with simvastatin at the same dose. As shown in Figures 1A,F, 50 μM simvastatin was identified as the maximum concentration that maintained >80% viability in both cell lines. This concentration was therefore chosen as the positive control dose. Meanwhile, it was also selected as the intermediate dose for *S*-HPABA, with 10 and 100 μM set as the low and high doses, respectively. The CCK-8 assay results indicate that *S*-HPABA at concentrations of 10-100 μM is within the safe and non-toxic range for both HepG2 and 3T3-L1 cells. Collectively, 10, 50 and 100 μM were selected for the cellular efficacy experiments.

The lipid-lowering efficacy of *S*-HPABA was evaluated in comparison to 50 μM simvastatin. Quantification of oil red O staining (Figures 1B,C,G–H) reveals abundant lipid droplets in the model groups of HepG2 and 3T3-L1 cells, with a 2.67-fold and 1.82-fold increase in lipid content compared to controls, respectively. *S*-HPABA dose-dependently attenuated lipid accumulation, with 50 μM achieving efficacy similar to simvastatin. Concordantly, TC and TG levels (Figures 1D,E,I,J) were elevated in model groups, validating successful establishment of the hyperlipidemic models. *S*-HPABA markedly decreased TC and TG in both models, with significant reductions at 10 μM in HepG2 cells and 50 μM in 3T3-L1 cells.

### 3.2 *S*-HPABA effectively inhibited high-fat diet (HFD)-induced lipid accumulation in zebrafish

Zebrafish, with their transparent larvae, offered a holistic view of pathology and were widely used in cardiovascular studies (37–40). This model allowed us to evaluate *S*-HPABA’s overall *in vivo* efficacy and avoid sampling bias inherent in localized tissue analysis.

In this study, the toxicity assessment revealed that *S*-HPABA had a favorable safety profile (Figures 2A–C). Specifically, embryo survival was unaffected at concentrations below 500 μM. In addition, no significant effects on survival, gross morphology, or heart rate were observed at concentrations up to 250 μM. Therefore, the pharmacodynamic testing doses were set within this safe range, with 250 μM selected as the high dose to evaluate the efficacy of *S*-HPABA at its maximum safe concentration. Ultimately, 10, 50 and 250 μM were chosen as the low, medium and high concentrations, respectively.

**Figure 2:**
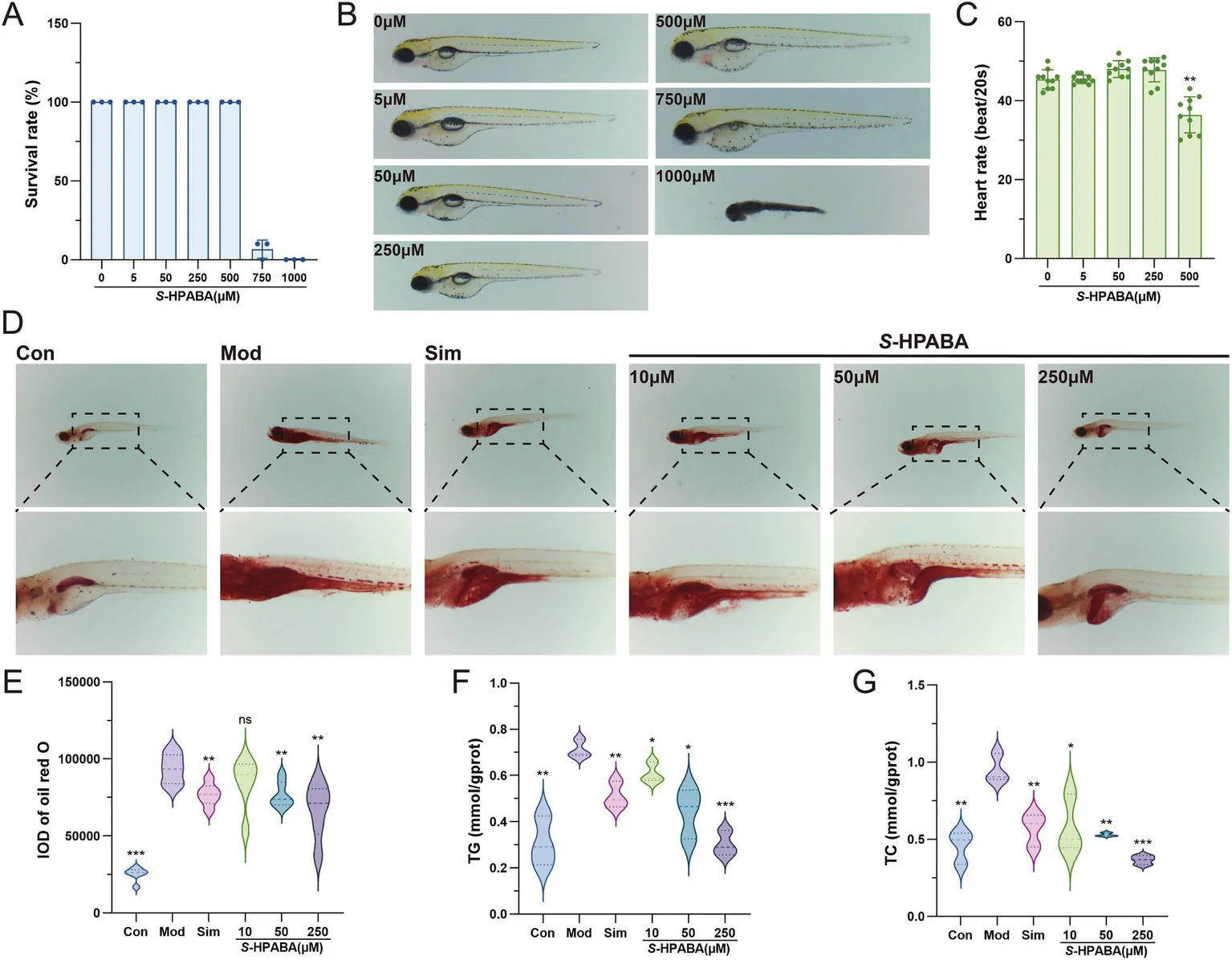
The toxicity and inhibitory abilities of *S*-HPABA on HFD-induced lipid accumulation in zebrafish. **(A,B)** The impact of *S*-HPABA on the survival rate and phenotype of zebrafish (*n* = 3). **(C)** Heart rate of zebrafish after exposure to *S*-HPABA (*n* = 10). **(D)** Oil red O staining of zebrafish after *S*-HPABA treatment. **(E)** The IOD of oil red O-stained lipid droplets (*n* = 6). **(F,G)** TG and TC levels in zebrafish samples after feeding with *S*-HPABA (*n* = 3). The data are presented as mean ± SD. *p < 0.05, **p < 0.01, ***p < 0.001 vs. the model group.

As shown in Figure 2D, the model group exhibits the most intense oil red O staining. Quantitative analysis using ImageJ revealed a significant increase in the model group compared to the control group, with a 3.71-fold higher intensity (Figure 2E). In contrast, *S*-HPABA greatly weakened oil red O staining intensity, with the most pronounced effect observed at 250 μM, which was 0.71 times of the model group. ImageJ-based quantification further demonstrated a dose-dependent decrease in IOD, indicating a reduction in lipid droplets in response to increasing *S*-HPABA concentrations.

In Figures 2F,G, the control group exhibits TC and TG levels of 0.46 and 0.31 mmol/gprot, respectively. The model group showed significantly elevated TC and TG concentrations, which increased to 0.95 and 0.71 mmol/gprot. All tested concentrations of *S*-HPABA substantially inhibited TC and TG disorders, showing a consistent trend with the IOD results. Notably, *S*-HPABA at 50 μM outperformed the positive control drug. Moreover, at 250 μM, *S*-HPABA achieved near-complete suppression of the abnormalities of TC and TG, with levels returning to 0.37 and 0.30 mmol/gprot, representing the optimal efficacy observed.

### 3.3 *S*-HPABA obviously alleviated HFD-induced lipid dysregulation

To uncover the lipid-lowering mechanism of *S*-HPABA, lipidomic analysis was performed on zebrafish samples from the control group, model group, and 250 μM *S*-HPABA-treated group.

#### 3.3.1 Stability assessment of the data

Data reliability was ensured by injecting a QC sample at intervals of six experimental runs. The PCA demonstrated excellent clustering of QC samples (Figures 3A,D) in both ionization modes, indicating highly stable experimental conditions throughout the analytical process. The total ion chromatogram (TIC) of the QC is shown in Supplementary Figure S2.

**Figure 3:**
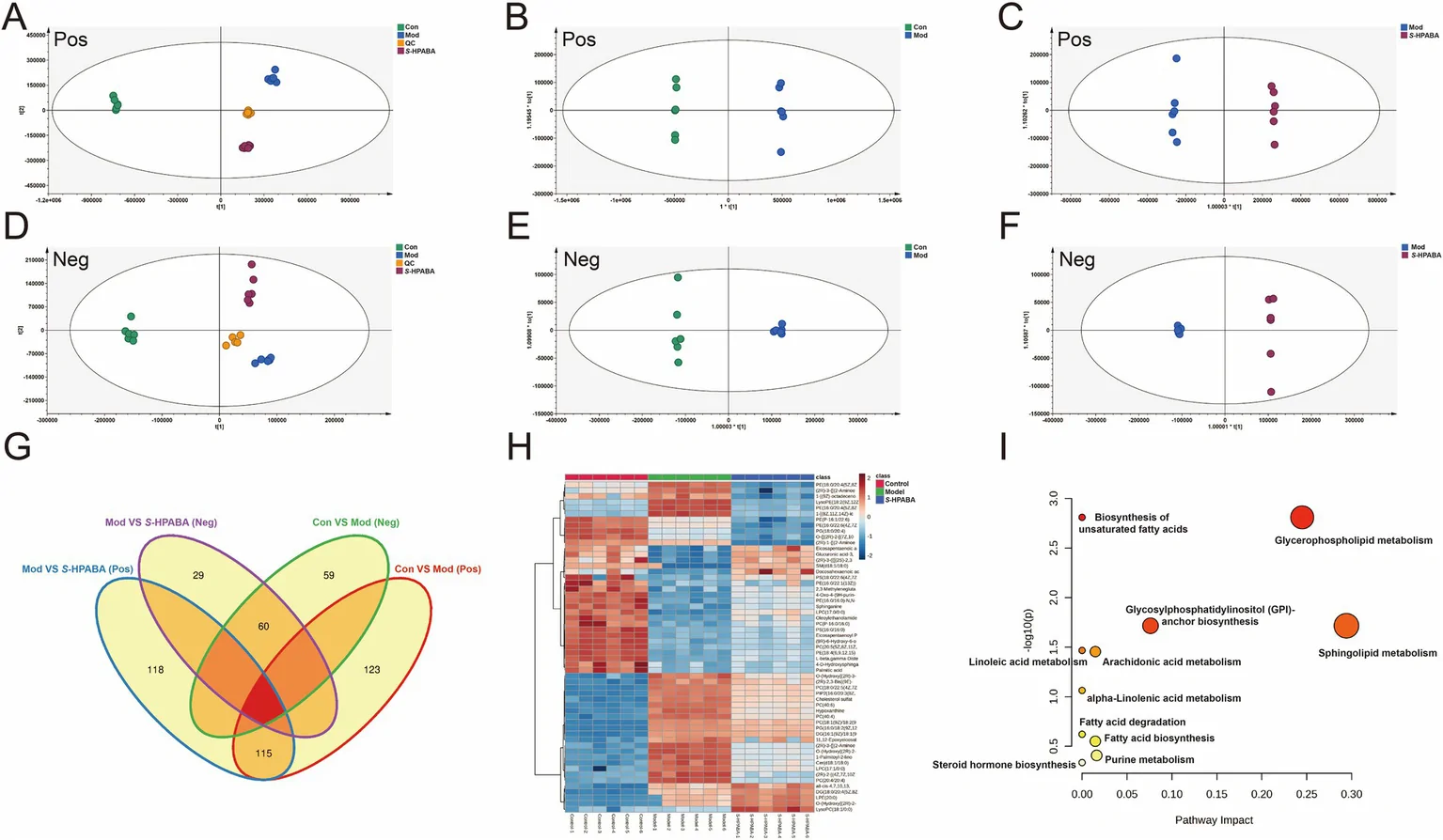
Corrective functions of *S*-HPABA on HFD-induced lipid metabolic disorders. **(A)** PCA plot in positive ion mode. **(B,C)** OPLS-DA analysis in positive ion environment. **(D)** Diagram of PCA in negative ion parameters. **(E,F)** OPLS-DA analysis in negative ion condition. **(G)** Venn diagram of differential ingredients in each group under positive and negative ion parameters. **(H)** Heatmap of metabolites with significant differences. **(I)** Pathway map enriched by differential components.

#### 3.3.2 Multivariate statistical analysis and differential metabolite screening

In our study, PCA was utilized to offer an initial overview of inter-group metabolic disparities and intra-group sample heterogeneity. Meanwhile, OPLS-DA was used for comparative analysis between two groups to identify differential metabolites. The PCA results presented in Figures 3A,D reveal distinct separation between groups and tight clustering within groups. Especially, the greatest distance was observed between the control and model groups, while the *S*-HPABA group clustered closer to the control, suggesting that *S*-HPABA treatment facilitates the restoration of lipid metabolism toward normal levels. The results of OPLS-DA demonstrate clear separation between different groups, indicating significant intergroup differences (Figure 3B,C,E,F). The fitting and predictive capabilities of the OPLS-DA model were assessed by R^2^ and Q^2^ values, respectively. The OPLS-DA model exhibited satisfactory performance with all parameters approaching 1 (Supplementary Table S2). Permutation test was conducted to ensure the OPLS-DA model was not overfitted. A model with Q^2^ less than 0 was considered valid. In Supplementary Figure S3, Supplementary Table S2, the Q^2^ values for the models were −0.465, −0.475, −0.533, −0.861, which were all in line with the requirements.

Subsequently, volcano plots and VIP plots were generated (Supplementary Figure S4) to screen for intergroup differential metabolites. Metabolites simultaneously satisfying the criteria of FC > 1.2 or < 0.8, *p*-value < 0.05, and VIP > 1 were chosen as potential candidates. Compounds that displayed significant differences in both the control vs. model and the model vs. *S*-HPABA comparison were defined as key differential metabolites. This yielded 115 and 60 metabolites in positive and negative modes, respectively (Figure 3G). After identification, 57 lipid metabolites were selected for further investigation (details provided in Supplementary Table S3).

#### 3.3.3 Analysis of differential metabolites and pathway enrichment

The 57 screened differential metabolites were primarily categorized into glycerophospholipids, lysophospholipids, sphingolipids, and fatty acids. Among these, glycerophospholipids were the most abundant category with 32 metabolites, followed by fatty acids with seven.

In order to visualize the impact of *S*-HPABA on the differential metabolites, a heatmap was drawn with MetaboAnalyst 6.0. In the heatmap, each column represents the abundance of metabolites in a single sample, while each row displays the relative abundance of an individual metabolite across various samples. Metabolite levels were color-coded, with red indicating higher abundance and blue suggesting lower. As shown in Figure 3H, compared to the model group, *S*-HPABA treatment resulted in the upregulation of 27 metabolites and the downregulation of 30 metabolites (details provided in Supplementary Table S3). Notably, the levels of 47 metabolites were reversed toward normal levels. For instance, over 70% of the glycerophospholipids in the treatment group showed a tendency to shift toward the control group’s profile. Simultaneously, *S*-HPABA intervention restored the abundance of six fatty acids close to those of control group. These findings reveal that *S*-HPABA can ameliorate lipid metabolic disorders and reduce HFD-induced lipid accumulation.

Differential metabolites were imported into the pathway analysis module of MetaboAnalyst 6.0 to further elucidate the metabolic pathways affected by *S*-HPABA. Figure 3I presents a total of 11 metabolic pathways, including biosynthesis of unsaturated fatty acids, glycerophospholipid metabolism, glycosylphosphatidylinositol (GPI) anchor biosynthesis, sphingolipid metabolism, linoleic acid metabolism, arachidonic acid metabolism, α-linolenic acid metabolism, fatty acid degradation, purine metabolism, fatty acid biosynthesis, and steroid hormone biosynthesis. In the pathway diagram, darker colors and larger bubbles represent greater pathway importance. Figure 3I reveals that glycerophospholipid metabolism was the most significantly enriched pathway, followed by biosynthesis of unsaturated fatty acids and sphingolipid metabolism.

### 3.4 Lipidomics combined with bioinformatics for screening candidate targets

When lipidomics integrated with bioinformatics, the potential targets generated by this combination embodied both the authenticity of omics and the comprehensive prediction ability of bioinformatics. Accordingly, this joint analysis strategy was adopted in this study to screen for the targets of *S*-HPABA. First, the differential lipids between the model group and treatment group in lipidomics were introduced into Cytoscape to build a “metabolite-reaction-enzyme-gene” network. In Figures 4A, 127 genes were closely related to 12 key metabolites and involved 50 reactions and 37 enzymes. These 127 genes would subsequently be subjected to joint analysis with bioinformatics.

**Figure 4:**
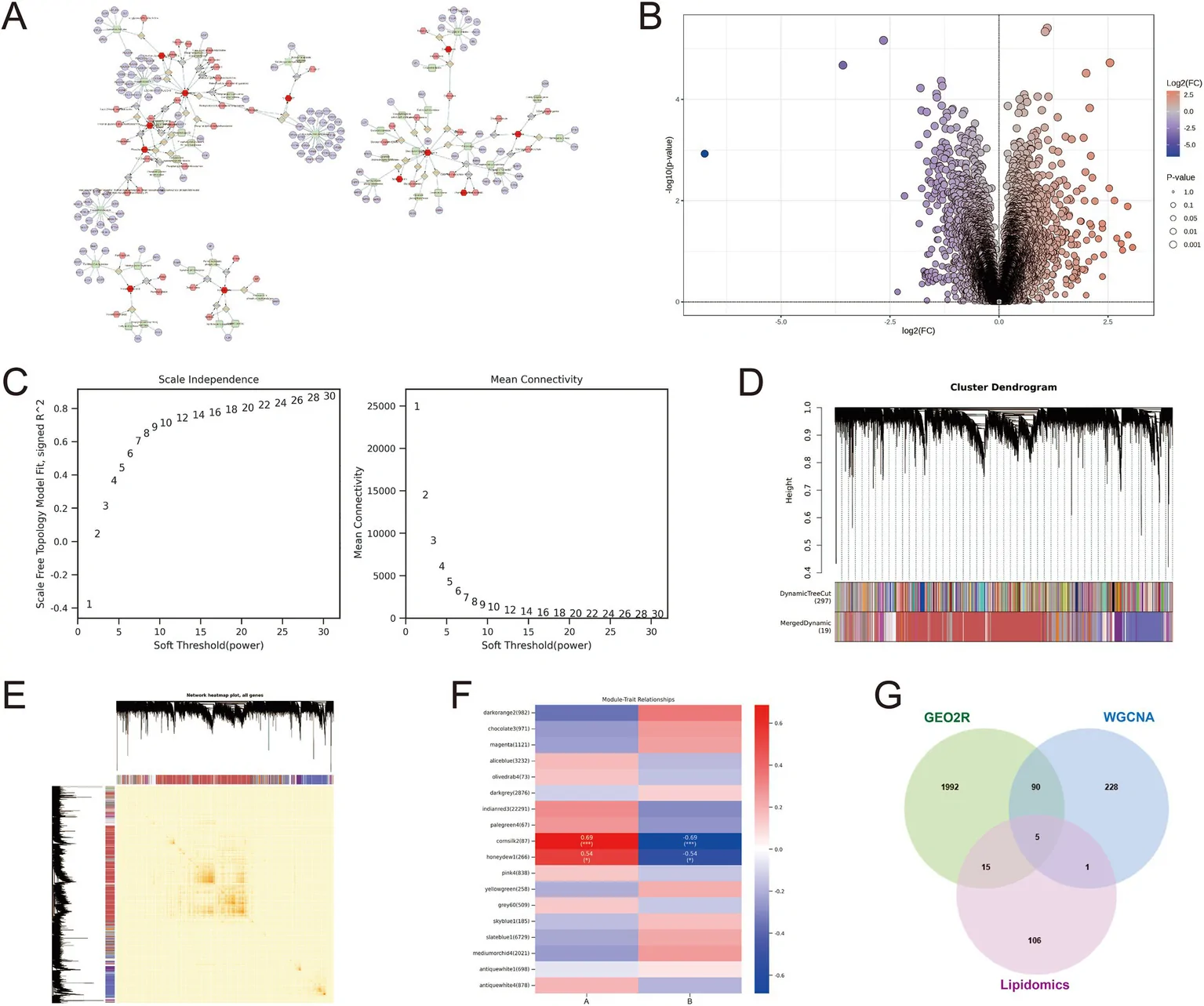
Target screening for the lipid-lowering effects of *S*-HPABA via lipidomics and bioinformatics. **(A)** The network of differential metabolite-reaction-enzyme-gene, where hexagons, diamonds, squares and circles represented metabolites, reactions, enzymes and genes, respectively. **(B)** Volcanic map of GEO2R. **(C)** The left figure presented the correlation coefficients under different soft thresholds, the picture in right depicted the average connectivity within networks created by different soft thresholds. **(D)** The upper section displayed a gene clustering dendrogram, and the lower described a gene module diagram. **(E)** Clustering heatmap of all genes. **(F)** Correlation heatmap of different modules and traits, with red indicating positive correlation and blue representing negative. **(G)** Venn diagram of lipidomics and bioinformatics analysis.

In bioinformatics analysis, the GSE88837 dataset, publicly available in the NCBI database, was analyzed in this study with both GEO2R and WGCNA algorithms to compute and screen obesity-related genes. As shown in the volcano plot (Figure 4B), a total of 5,143 upregulated genes and 4,857 downregulated genes were distinguished by GEO2R. 2,102 differentially expressed genes were obtained based on the criteria of *P* < 0.05 and |LogFC| > 0.5.

Subsequently, WGCNA analysis was performed to gain gene modules highly related with obesity in the GSE88837 dataset. It was essential to determine an appropriate soft threshold for the adjacency matrix, which was beneficial for constructing a better scale-free network. The network correlation coefficients and average connectivity for different soft thresholds were determined in Figure 4C. The left portion of the figure illustrated the correlation coefficients for different soft thresholds, while the right side depicted the average connectivity of networks constructed using the same soft thresholds. When the parameter was set to 0.8, 24 represented the minimum soft threshold for constructing a scale-free network, at which point both the correlation coefficient and average connectivity of the network reached the optimal level. This soft threshold was used to build a weighted co-expression network model, which was then used to generate a gene clustering tree (Figure 4D). The upper half of Figure 4D displayed correlations among thousands of genes, and the lower half indicated distinct gene modules. Dynamic tree cut represented the initial coarse segmentation results, and merged dynamic indicated the outcomes after optimization. This study ultimately divided the genes in the GSE88837 dataset into 19 distinct modules. Figure 4E was the clustering heatmap of all genes. Pearson analysis was used to calculate the correlation coefficients and *p*-values between gene modules and traits. The results suggested that the *p*-values for the cornsilk2 and honeydew1 modules met the significance threshold (Figure 4F). Following the integration of these two modules (87 and 266 genes each) and elimination of redundant entries, 324 genes were selected for subsequent analysis.

Genes that simultaneously existed in GEO2R, WGCNA and lipidomics were considered as candidate targets of *S*-HPABA. In Figure 4G, there were five intersection genes, namely FASN, TYMP, PLA2G2A, ALG2, and SMPD3.

### 3.5 Validation of the core targets with RT-qPCR and western blot tests

In order to define the lipid-lowering targets of *S*-HPABA, the five candidate genes acquired from the previous step were validated by RT-qPCR. And then the protein expression levels of the targets were measured by western blot analysis.

Figures 5A–E illustrate that the mRNA levels of FASN, TYMP, PLA2G2A, ALG2 and SMPD3 all exhibited visible changes between the control group and model group. But the dysregulation of SMPD3 and ALG2 has not been alleviated after treatment with either simvastatin or *S*-HPABA. In contrast, drug intervention dose-dependently suppressed the transcription of FASN, TYMP, and PLA2G2A, as evidenced by evidently reduced mRNA levels in the medium- and high-dose groups compared to the model group. The western blot results are consistent with the RT-qPCR data, implying that all tested doses of *S*-HPABA attenuate the upregulation of FASN, TYMP and PLA2G2A provoked by sodium oleate (Figures 5F–I). Among these, *S*-HPABA exhibited the most pronounced effect on FASN, sharply inhibiting its production even at 10 μM. The influences on PLA2G2A and TYMP were comparatively moderate, with significant regulatory effects observed at doses of 50 μM and 100 μM, respectively.

**Figure 5:**
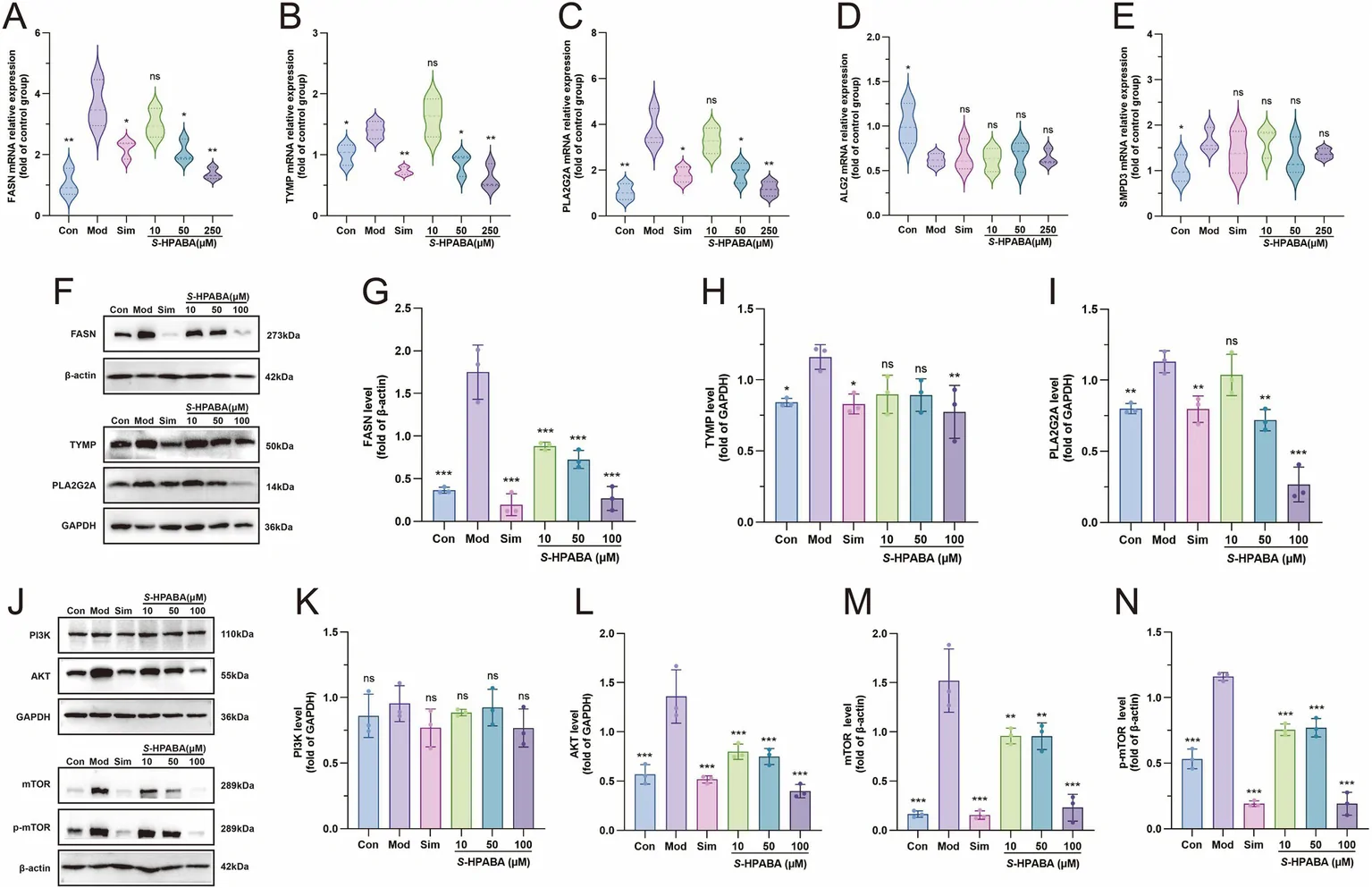
Validation of key targets by RT-qPCR and western blotting. **(A–E)** Effects of *S*-HPABA on the mRNA levels of FASN, TYMP, PLA2G2A, ALG2, and SMPD3 in Zebrafish. **(F–I)** Influences of *S*-HPABA on the protein expression of FASN, TYMP and PLA2G2A in HepG2 cells. **(J–N)** Impact of *S*-HPABA on the PI3K/AKT/mTOR signaling pathway in HepG2 cells. The data are presented as mean ± SD (*n* = 3). *p < 0.05, **p < 0.01, ***p < 0.001 vs. the model group.

The PI3K/AKT/mTOR signaling pathway was highly correlated with FASN generation. The activation of this pathway promoted the binding of SREBP-1C to the FASN gene promoter, thereby enhancing FASN transcription and synthesis (41, 42). To thoroughly validate the regulatory role of *S*-HPABA on FASN, this pathway was assessed by western blot analysis in this experiment. In Figures 5J–N, the expression levels of PI3K, AKT, mTOR and p-mTOR are clearly elevated by sodium oleate, while these markers are markedly reduced following intervention with simvastatin or *S*-HPABA. It was noteworthy that *S*-HPABA evinced a clear dose–response relationship, and its potency at matched concentrations rivaled that of simvastatin. Collectively, these results indicate that *S*-HPABA alleviates metabolic disorders by suppressing the activation of the PI3K/AKT/mTOR pathway and reducing lipid biosynthesis.

## 4 Discussion

### 4.1 The structure of *S*-HPABA implicates a potential in lipid lowering

Our earlier studies have found that *S*-HPABA, a natural structural analog of aspirin, possesses excellent anti-inflammatory and antithrombotic activities. As shown in Supplementary Figure S5, *S*-HPABA and aspirin share a common benzoic acid structure but differ in their side chains: acetyl group in aspirin versus 2-hydroxypropionamide in *S*-HPABA. Pharmacodynamic studies indicate that *S*-HPABA exerts antithrombotic effect through the inhibition of platelet aggregation, which is align with aspirin. In contrast, *S*-HPABA demonstrated a markedly improved gastrointestinal safety profile compared to aspirin. These results suggest that *S*-HPABA and aspirin share similar pharmacological activities, while *S*-HPABA shows a better safety profile.

The emerging evidence indicates that aspirin effectively diminishes liver fat accumulation, underscoring its potential as a lipid-lowering agent (43). Based on our prior evidence, *S*-HPABA has been presumed to possess lipid-lowering properties. The present results confirm this hypothesis, demonstrating that *S*-HPABA exhibits outstanding lipid-lowering activity along with a favorable safety profile. These findings position *S*-HPABA as a promising nutraceutical candidate for future applications in metabolic health.

### 4.2 *S*-HPABA alleviates HLP by correcting lipid dysregulation

The 57 differential metabolites affected by *S*-HPABA were closely associated with critical biological processes such as signal transduction, energy storage, and inflammation regulation (44–46). *S*-HPABA exerted lipid-lowering effects by modulating the levels of these lipid metabolites. Furthermore, glycerophospholipid metabolism, biosynthesis of unsaturated fatty acids and sphingolipid metabolism played crucial roles in lipid-lowering effects. Liupao tea (LPT) ameliorated lipid metabolic disorders in obese mice through glycerophospholipid metabolism (47), while epigallocatechin gallate (EGCG) effectively mitigated HFD-induced HLP by regulating the same pathway (48). Among the *S*-HPABA-regulated glycerophospholipid metabolites, six PCs, two PSs, two PEs, and three LysoPCs were enriched in glycerophospholipid metabolism, indicating that this pathway is central to the lipid-lowering efficacy of *S*-HPABA.

Additionally, the biosynthesis of unsaturated fatty acids and sphingolipid metabolism also played crucial roles in preventing and treating lipid metabolic disorders. It was reported that *Bacteroides thetaiotaomicron* could enhance unsaturated fatty acid biosynthesis, which constituted a key mechanism for regulating lipid metabolism and alleviating non-alcoholic fatty liver disease (49). Ginkgolide B, a potential anti-hyperlipidemic agent, mitigated HFD-induced metabolic inflammation and oxidative stress by rectifying disruptions in sphingolipid metabolism (50). Consistent with the reports, *S*-HPABA prominently reversed the decline of polyunsaturated fatty acids in the model group. Eicosapentaenoic acid and docosahexaenoic acid were both enriched in the biosynthesis of unsaturated fatty acids. Concurrently, *S*-HPABA restored the concentrations of pivotal sphingolipids (e.g., sphinganine, SM(d18:1/18:0), Cer(d18:1/18:0), and 4-D-hydroxysphinganine) to levels of control group, implying a normalization of sphingolipid metabolism.

### 4.3 *S*-HPABA exerts pharmacological activity by modulating the expression of FASN, TYMP and PLA2G2A

It is well known that HLP has multifactorial etiology, involving genetic susceptibility, dietary patterns, and other factors. Among these, obesity resulting from a HFD stands as one of the most prevalent and significant risk factors (51, 52). In the present study, bioinformatic analysis was designed to investigate the impact of HFD on gene expression profiles. Therefore, we intentionally excluded datasets involving familial hypercholesterolemia and instead selected obesity-related datasets to avoid the dominant influence of monogenic mutations on transcriptional profiles. This provided more suitable data for target screening strategies integrated with lipidomics.

In this study, five genes were selected through a combined approach of lipidomics and bioinformatics. Details of the metabolites and pathways closely associated with these genes are presented in Supplementary Table S4. Following validation by RT-qPCR and western blotting, FASN, PLA2G2A and TYMP were identified as the primary targets of *S*-HPABA. These genes undertook critical roles in lipid synthesis and metabolism, with FASN serving as a central and rate-limiting enzyme in lipogenesis. FASN’s overexpression was a primary driver of excessive triglyceride synthesis and accumulation, directly contributing to the pathogenesis of obesity, HLP, fatty liver disease, and other related metabolic disorders (53, 54). Inhibiting FASN expression was a common approach to alleviating high-fat conditions. As reported, fuzhuan brick tea mitigated fatty liver in obese mice by diminishing FASN content (55). Similarly, peptides derived from *Harpadon nehereus* bone ameliorate sodium palmitate-induced lipid accumulation in cells through downregulation of FASN (56).

Hydrolysis of phospholipids was the primary action of PLA2G2A, which released pro-inflammatory factors such as arachidonic acid. As a result, its lipid-lowering effects were frequently associated with anti-inflammatory responses. A notable example was green tea catechins, which downregulated PLA2G2A expression via the glycerophospholipid metabolism pathway (57). This could disrupt the differentiation process of 3T3-L1 cells, thereby exerting anti-obesity and anti-inflammatory abilities. Although TYMP exhibited slightly weaker activity than FASN and PLA2G2A, it served as an important indicator for diagnosing fatty liver disease. For instance, researchers discovered that fatty liver tissue contained more TYMP than normal liver tissue (58). These existing findings were aligned with the results of this study, suggesting that *S*-HPABA exerted the lipid-lowering effects through a multi-target synergistic mechanism, with FASN being the predominant target.

### 4.4 *S*-HPABA suppresses lipid accumulation by inhibiting the PI3K/AKT/mTOR signaling pathway

The PI3K/AKT/mTOR pathway played a crucial role in the production of FASN. As depicted in Figure 6 (59), there existed a close relationship among them. The increased exogenous fat from a HFD led to insulin resistance, which elevated insulin levels in the body (60). Insulin acted as an activator of the PI3K/AKT signaling pathway. The activated signaling pathways increased the levels and activity of mTOR, subsequently boosting the expression of the transcription factor SREBP-1C (61). The transcription of FASN was initiated by SREBP-1C, which generated abundant mRNA transcripts and ultimately elevated FASN protein abundance.

**Figure 6:**
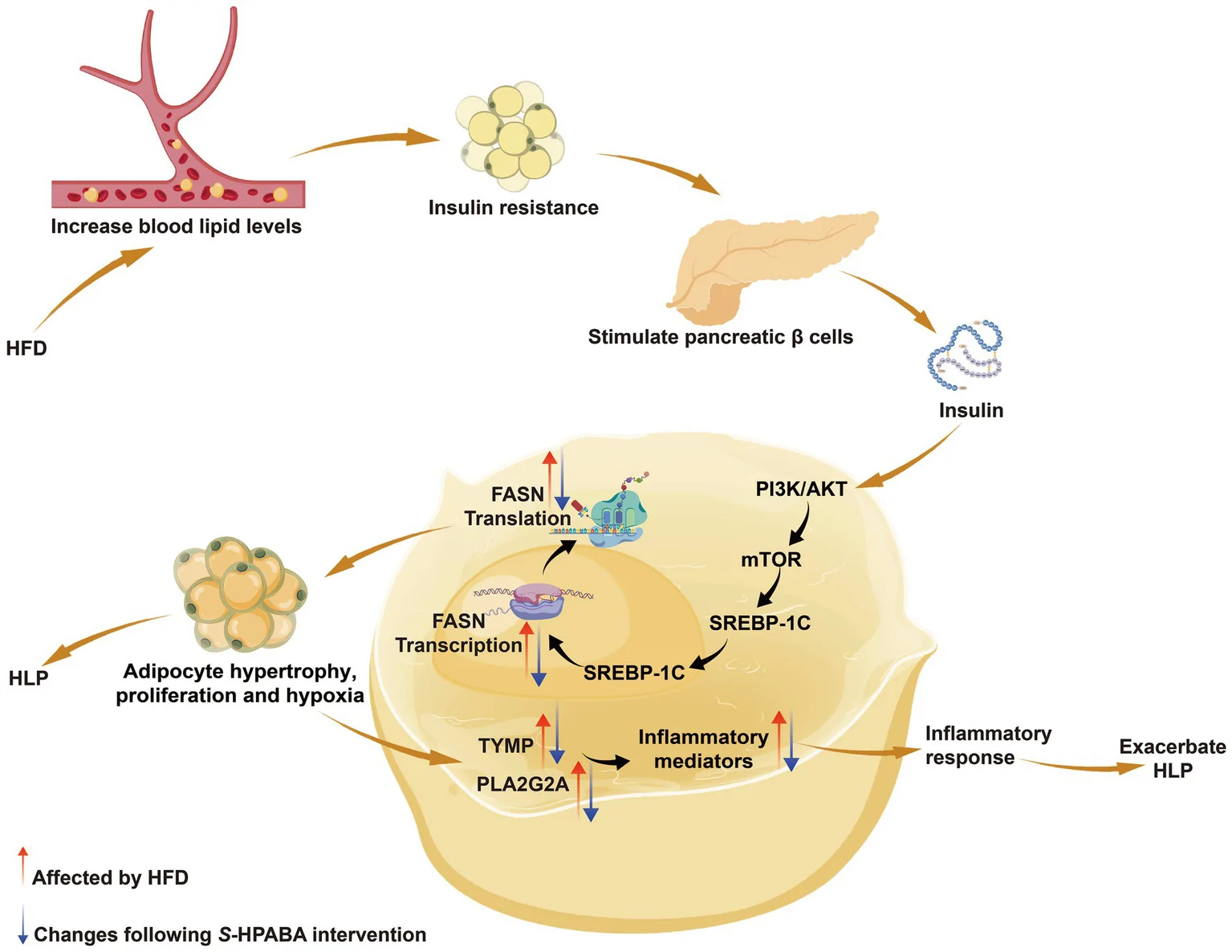
Mechanism of *S*-HPABA in alleviating HLP. In the diagram, red arrows represented upregulation induced by a HFD, while blue arrows indicated downregulation mediated by *S*-HPABA.

The elevated FASN expression induced lipid accumulation and subsequent adipocyte hypertrophy. To alleviate hypoxia in hypertrophic adipocytes, the expression of TYMP was upregulated to promote angiogenesis in adipose tissue (62). However, 2-deoxy-D-ribose-1-phosphate (2-dR-1P), the enzymatic product of TYMP, recruited macrophages and thereby stimulated the release of inflammatory cytokines (63). This process transformed simple fat storage into a pathological metabolic disorder. Simultaneously, the transcription factor of PLA2G2A was activated by elevated fatty acid caused by FSAN abnormalities (64). Through its hydrolysis of phospholipids, PLA2G2A generated potent lipid mediators (e.g., prostaglandins, leukotrienes) that amplify tissue inflammation in obesity, thereby driving the progression toward metabolic disorders.

By blocking the PI3K/AKT/mTOR pathway activation, *S*-HPABA suppressed the expression of key lipid metabolic genes, effectively breaking the pathological cycle driven by HFD and resulting in HLP mitigation.

### 4.5 Limitation

Although the results of this study, together with the existing literature, provide strong evidence for a causal relationship between the PI3K/AKT/mTOR pathway and FASN, the evidence remains indirect (65–67). Direct verification of this causality in *S*-HPABA-mediated lipid lowering would require PI3K/AKT/mTOR inhibitors (such as LY294002 and rapamycin) to confirm whether pathway inhibition is indeed responsible for FASN downregulation. In future studies, mouse models will be used to further validate the effects of *S*-HPABA, and pathway inhibitors will be employed to verify the causal relationship between the PI3K/AKT/mTOR pathway and FASN.

## 5 Conclusion

This study demonstrates that *S*-HPABA is a promising bioactive compound characterized by a favorable safety profile and potent lipid-lowering efficacy. *S*-HPABA alleviates HFD-induced lipid metabolic disorders through multiple pathways. It exertes lipid-lowering effects via synergistic multi-target regulation (including FASN, TYMP and PLA2G2A), with FASN emerging as the most critical target. Mechanistically, *S*-HPABA-mediated inhibition of the PI3K/AKT/mTOR pathway may contribute to the downregulation of FASN at both the transcriptional and translational levels. Ultimately, it attenuates lipid synthesis and accumulation, thereby suppressing the progression of HLP. This study not only provides a low-toxicity and high-efficiency bioactive compound for HLP prevention and management, but also offers a new template and perspective for future marine resource development in the context of metabolic health.

## Data Availability

The original contributions presented in the study are included in the article/Supplementary material, further inquiries can be directed to the corresponding authors.

## References

[1] Chen YT Li KP Zhao H Hao ZS Yang YX Gao MM . Integrated lipidomics and network pharmacology analysis to reveal the mechanisms of berberine in the treatment of hyperlipidemia. J Transl Med. (2022) 20:1. doi: 10.1186/s12967-022-03623-0, 36076294 PMC9461205

[2] Xiang Y Mao L Zuo ML Song GL Tan LM Yang ZB. The role of microRNAs in hyperlipidemia: from pathogenesis to therapeutical application. Mediat Inflamm. (2022) 2022:3101900. doi: 10.1155/2022/3101900, 35757107 PMC9232323

[3] Yao YS Li TD Zeng ZH Li TD. Mechanisms underlying direct actions of hyperlipidemia on myocardium: an updated review. Lipids Health Dis. (2020) 19:1. doi: 10.1186/s12944-019-1171-8, 32035485 PMC7007679

[4] Shi LJ Tang XW He J Shi WB. Genetic evidence for a causal relationship between hyperlipidemia and type 2 diabetes in mice. Int J Mol Sci. (2022) 23:6184. doi: 10.3390/ijms23116184, 35682864 PMC9181284

[5] Cheng DY Zhang M Zheng YZ Wang M Gao YL Wang XD . α-Ketoglutarate prevents hyperlipidemia-induced fatty liver mitochondrial dysfunction and oxidative stress by activating the AMPK-pgc-1α/Nrf2 pathway. Redox Biol. (2024) 74:103230. doi: 10.1016/j.redox.2024.103230 PMC11226981 38875959

[6] Wereski R Kimenai DM Bularga A Taggart C Lowe DJ Mills NL . Risk factors for type 1 and type 2 myocardial infarction. Eur Heart J. (2022) 43:127–35. doi: 10.1093/eurheartj/ehab581, 34431993 PMC8757580

[7] Zhang ZQ Wu HY Wang T Liu Y Meng C. Mechanisms of myocardial damage due to hyperlipidemia: a review of recent studies. Med Sci Monit. (2022) 28:937051. doi: 10.12659/msm.937051, 36110037 PMC9487343

[8] Stachowicz A Czepiel K Wiśniewska A Stachyra K Ulatowska Białas M Kuśnierz Cabala B . Mitochondria-targeted hydrogen sulfide donor reduces fatty liver and obesity in mice fed a high fat diet by inhibiting de novo lipogenesis and inflammation via mTOR/SREBP-1 and NF-κB signaling pathways. Pharmacol Res. (2024) 209:107428. doi: 10.1016/j.phrs.2024.107428 39303773

[9] Giles LA. Hyperlipidemia prevention and management utilizing lifestyle changes. J Midwifery Womens Health. (2024) 69:361–9. doi: 10.1111/jmwh.13637, 38678434

[10] Anderson TJ Grégoire J Pearson GJ Barry AR Couture P Dawes M . 2016 Canadian cardiovascular society guidelines for the management of dyslipidemia for the prevention of cardiovascular disease in the adult. Can J Cardiol. (2016) 32:1263–82. doi: 10.1016/j.cjca.2016.07.510, 27712954

[11] Kirsten BD Grossman DC Curry SJ Davidson KW Epling JW García FAR . Statin use for the primary prevention of cardiovascular disease in adults. JAMA. (2016) 316:19. doi: 10.1001/jama.2016.15450 27838723

[12] Singh S Zahoor I Sharma N Behl T Kanojia N Sehgal A . Insights into the pivotal role of statins and its nanoformulations in hyperlipidemia. Environ Sci Pollut Res. (2022) 29:76514–31. doi: 10.1007/s11356-022-23043-3, 36161571

[13] Husain MJ Spencer G Nugent R Kostova D Richter P. The cost-effectiveness of hyperlipidemia medication in low- and middle-income countries: a review. Glob Heart. (2022) 17:18. doi: 10.5334/gh.1097, 35342693 PMC8896253

[14] Ye JH Li L Hu ZX Xu BJ. Exploring the molecular mechanism of action of yinchen wuling powder for the treatment of hyperlipidemia, using network pharmacology, molecular docking, and molecular dynamics simulation. Biomed Res Int. (2021) 2021:1. doi: 10.1155/2021/9965906 PMC8568510 34746316

[15] Zeng WW Deng H Luo YN Zhong SL Huang M Tomlinson B. Advances in statin adverse reactions and the potential mechanisms: a systematic review. J Adv Res. (2024) 76:781–97. doi: 10.1016/j.jare.2024.12.020, 39681285 PMC12793736

[16] Wang BJ Huang S Li SQ Deng YQ Li ZY Wang YZ . Hepatotoxicity of statins: a real-world study based on the US food and drug administration adverse event reporting system database. Front Pharmacol. (2025) 15:1502791. doi: 10.3389/fphar.2024.1502791, 39840096 PMC11747658

[17] Murphy WA Lin N Damask A Schwartz GG Steg PG Szarek M . Pharmacogenomic study of statin-associated muscle symptoms in the ODYSSEY OUTCOMES trial. Circ Genom Precis Med. (2022) 15:3. doi: 10.1161/circgen.121.003503 PMC9213083 35543701

[18] Tsui L Chen LW Ye PY Xu SL Wu SJ Chen SCI . Adverse drug reactions of non-statin antihyperlipidaemic drugs in China from 1989 to 2019: a national database analysis. BMJ Open. (2023) 13:5. doi: 10.1136/bmjopen-2022-068915, 37253501 PMC10254877

[19] Fang YW She JL Zhang X Gu TW Xie DN Luo XW . Discovery of anti-hypercholesterolemia agents targeting LXRα from marine microorganism-derived natural products. J Nat Prod. (2024) 87:322–31. doi: 10.1021/acs.jnatprod.3c01029 38334086

[20] Lv L Maimaitiming M Xia SL Yang JC Zhang TT Wang YM . MR2938 relieves DSS-induced colitis in mice through inhibiting NF-κB signaling and improving epithelial barrier. Mar Life Sci Technol. (2025) 25:25. doi: 10.1007/s42995-025-00285-x PMC12662929 41322271

[21] Haque N Parveen S Tang TT Wei JE Huang ZN. Marine natural products in clinical use. Mar Drugs. (2022) 20:528. doi: 10.3390/md20080528, 36005531 PMC9410185

[22] Ren XH Xie XY Chen BX Liu L Jiang CQ Qian Q. Marine natural products: a potential source of anti-hepatocellular carcinoma drugs. J Med Chem. (2021) 64:7879–99. doi: 10.1021/acs.jmedchem.0c02026, 34128674

[23] Li YR Li GJ Feng JW Li SL Liu N. Advances in research on marine natural products for modulating the inflammatory microenvironment. Phytother Res. (2025) 39:1238–58. doi: 10.1002/ptr.8418, 39844461

[24] Al Rijjal D Liu Y Lai M Song YC Danaei Z Wu A . Vascepa protects against high-fat diet-induced glucose intolerance, insulin resistance, and impaired β-cell function. iScience. (2021) 24:102909. doi: 10.1016/j.isci.2021.102909, 34458694 PMC8379293

[25] Lin JP Chen SW Butt UD Yan M Wu B. A comprehensive review on ziconotide. Heliyon. (2024) 10:e31105. doi: 10.1016/j.heliyon.2024.e31105, 38779019 PMC11110537

[26] Marrero RJ Shastri VM Nicolet D Mrózek K Walker CJ Blum WG . Cytarabine pharmacogenomics and outcomes among children and young adults with acute myeloid leukemia. JAMA Netw Open. (2025) 8:6. doi: 10.1001/jamanetworkopen.2025.16296, 40549387 PMC12186577

[27] Jan N Shah H Khan S Nasar F Madni A Badshah SF . Old drug, new tricks: polymer-based nanoscale systems for effective cytarabine delivery. Naunyn Schmiedeberg's Arch Pharmacol. (2023) 397:3565–84. doi: 10.1007/s00210-023-02865-z, 38015258

[28] Nakamura T Sudo A. The role of trabectedin in soft tissue sarcoma. Front Pharmacol. (2022) 13:777872. doi: 10.3389/fphar.2022.777872, 35281940 PMC8904719

[29] Zhang QL Wang DL Zhang MY Zhao YL Yu ZG. Studies on new activities of enantiomers of 2-(2-hydroxypropanamido) benzoic acid: antiplatelet aggregation and antithrombosis. PLoS One. (2017) 12:e0170334. doi: 10.1371/journal.pone.0170334, 28107496 PMC5249173

[30] Rong R Zhang RZ Wang X Dan YH Zhao YL Yu ZG. Synthesis, pharmacological evaluation and molecular docking of novel R-/S-2-(2-hydroxypropanamido)-5-trifluoromethyl benzoic acid as dual anti-inflammatory anti-platelet aggregation agents. Naunyn Schmiedeberg's Arch Pharmacol. (2019) 393:967–78. doi: 10.1007/s00210-019-01753-9, 31802171

[31] Wang JJ Zhao YL An ZZ Sun TM Yu ZG. Synthesis and contents determination of the related substances of 2-lactoylaminobenzoic acid by RP-HPLC. J Shenyang Pharm Univ. (2014) 31:1. doi: 10.14066/j.cnki.cn21-1349/r.2014.01.018

[32] Teixeira FS Pimentel LL Vidigal SSMP Azevedo Silva J Pintado ME Rodríguez Alcalá LM. Differential lipid accumulation on HepG2 cells triggered by palmitic and linoleic fatty acids exposure. Molecules. (2023) 28:5. doi: 10.3390/molecules28052367 PMC10005272 36903612

[33] Zhao YS Tong XM Wu XM Bai J Fan ST Zhu Y . Metabolomics reveal the regulatory effect of polysaccharides from fermented barley bran extract on lipid accumulation in HepG2 cells. Meta. (2023) 13:2. doi: 10.3390/metabo13020223, 36837842 PMC9962758

[34] Monmai C Kim JS Baek SH. Germinated rice seeds improved resveratrol production to suppress adipogenic and inflammatory molecules in 3T3-L1 adipocytes. Molecules. (2023) 28:15. doi: 10.3390/molecules28155750, 37570719 PMC10420918

[35] Soong TC Lee KT Hsu YC Tsai TH. Cucurbitacin E suppresses adipogenesis and lipid accumulation in 3T3-L1 adipocytes without cytotoxicity. Biomedicine. (2025) 13:8. doi: 10.3390/biomedicines13081826, 40868081 PMC12383856

[36] Shihana F Cholan PM Fraser S Oehlers SH Seth D. Investigating the role of lipid genes in liver disease using fatty liver models of alcohol and high fat in zebrafish (*Danio rerio*). Liver Int. (2023) 43:2455–68. doi: 10.1111/liv.15716, 37650211

[37] Zhao DP Chen XQ Wang RQ Pang HQ Wang JB Liu L. Determining the chemical profile of *Caragana jubata* (pall.) Poir. By UPLC–QTOF–MS analysis and evaluating its anti-ischemic stroke effects. J Ethnopharmacol. (2023) 309:116275. doi: 10.1016/j.jep.2023.116275, 36806344

[38] Zhu H Lan CH Zhao D Wang N Du D Luo HB . Wuliangye baijiu but not ethanol reduces cardiovascular disease risks in a zebrafish thrombosis model. NPJ Sci Food. (2022) 6:1. doi: 10.1038/s41538-022-00170-2, 36470888 PMC9723178

[39] Wang LW Cui XY He JF Duan S Liu CR Shan CB . Hydroxysafflor yellows alleviate thrombosis and acetaminophen-induced toxicity in vivo by enhancing blood circulation and poison excretion. Phytomedicine. (2021) 87:153579. doi: 10.1016/j.phymed.2021.153579, 33991865

[40] Rao C Hu RX Hu YX Jiang Y Zou X Tang HL . Theoretical exploring of potential mechanisms of antithrombotic ingredients in danshen-chishao herb-pair by network pharmacological study, molecular docking and zebrafish models. Chin Med. (2024) 19:1. doi: 10.1186/s13020-024-00970-6, 39014502 PMC11253416

[41] Wang X Huang MJ Lu YX Li L Chen WY Wu QL . Integrated metabolomics and network pharmacology reveal the PI3K/Akt-mediated therapeutic mechanism of *Abrus cantoniensis* in lipid metabolism disorders. Phytomedicine. (2025) 145:156953. doi: 10.1016/j.phymed.2025.156953, 40517619

[42] Chen T Wang TT Shi YX Deng J Yan X Zhang CB . Integrated network pharmacology, metabolomics and molecular docking analysis to reveal the mechanisms of quercetin in the treatment of hyperlipidemia. J Pharm Biomed Anal. (2025) 252:116507. doi: 10.1016/j.jpba.2024.116507 39383544

[43] Simon TG Wilechansky RM Stoyanova S Grossman A Dichtel LE Lauer GM . Aspirin for metabolic dysfunction-associated steatotic liver disease without cirrhosis. JAMA J Am Med Assoc. (2024) 331:920. doi: 10.1001/jama.2024.1215, 38502074 PMC10951738

[44] Yang SY Wei ZJ Luo JC Wang XC Chen GH Guan X . Integrated bioinformatics and multiomics reveal Liupao tea extract alleviating NAFLD via regulating hepatic lipid metabolism and gut microbiota. Phytomedicine. (2024) 132:155834. doi: 10.1016/j.phymed.2024.155834, 38941818

[45] Liu DM Wang JH Zeng HL Zhou F Wen BB Zhang XN . The metabolic regulation of Fuzhuan brick tea in high-fat diet-induced obese mice and the potential contribution of gut microbiota. Food Funct. (2022) 13:356–74. doi: 10.1039/d1fo02181h, 34904994

[46] Zhi DD He XY Yang LF Xue YF Liu YQ Yue D . Royal jelly acid alleviates diet-induced hyperlipidemia through regulation of oxidative stress and tryptophan metabolism. Eur J Pharmacol. (2025) 998:177500. doi: 10.1016/j.ejphar.2025.177500, 40086581

[47] Zhou HL Wang XC She ZY Huang L Wei HJ Yang SY . Combining bioinformatics and multiomics strategies to investigate the key microbiota and active components of liupao tea ameliorating hyperlipidemia. J Ethnopharmacol. (2024) 333:118438. doi: 10.1016/j.jep.2024.118438, 38848972

[48] Wen JJ Li MZ Chen CH Hong T Yang JR Huang XJ . Tea polyphenol and epigallocatechin gallate ameliorate hyperlipidemia via regulating liver metabolism and remodeling gut microbiota. Food Chem. (2023) 404:134591. doi: 10.1016/j.foodchem.2022.134591, 36444016

[49] Li H Wang XK Tang M Lei L Li JR Sun H . *Bacteroides thetaiotaomicron* ameliorates mouse hepatic steatosis through regulating gut microbial composition, gut-liver folate and unsaturated fatty acids metabolism. Gut Microbes. (2024) 16:1. doi: 10.1080/19490976.2024.2304159, 38277137 PMC10824146

[50] Liu Y Wang F Xu HY Wang HX Lu ML Cheng L. Ginkgolide B attenuates hyperlipidemia by restoring sphingolipid homeostasis and activating PPARα and Nrf2 pathways. Sci Rep. (2025) 15:28774. doi: 10.1038/s41598-025-14626-4, 40770408 PMC12329028

[51] Chai ZY Yan YH Zan SY Meng XH Zhang F. Probiotic-fermented blueberry pomace alleviates obesity and hyperlipidemia in high-fat diet C57BL/6J mice. Food Res Int. (2022) 157:111396. doi: 10.1016/j.foodres.2022.111396 35761652

[52] Zhang YH Yao YJ. The association between obesity indicators and mortality among individuals with hyperlipidemia: evidence from the NHANES 2003–2018. Lipids Health Dis. (2025) 24:1. doi: 10.1186/s12944-025-02442-8, 39856680 PMC11761752

[53] Chen YS Zheng KD Leng Y Zhang Z Li XL Li XY . Alleviating effect of *Lactobacillus fermentum* E15 on hyperlipidemia and hepatic lipid metabolism in zebrafish fed by a high-fat diet through the production of short-chain fatty acids. Front Nutr. (2025) 12:1522982. doi: 10.3389/fnut.2025.1522982, 40098735 PMC11911183

[54] Meade R Ibrahim D Engel C Belaygorod L Arif B Hsu F-F . Targeting fatty acid synthase reduces aortic atherosclerosis and inflammation. Commun Biol. (2025) 8:262. doi: 10.1038/s42003-025-07656-1, 39972116 PMC11840040

[55] Liu D Wang S Liu Y Luo Y Wen B Wu W . Fuzhuan brick tea ameliorates hepatic steatosis and steatohepatitis through gut microbiota-derived aryl hydrocarbon receptor ligands in high-fat diet-induced obese mice. Food Funct. (2023) 14:8351–68. doi: 10.1039/d3fo01782f, 37606634

[56] Song SY Zhao W Lin QX Pei JF Jin HX. Peptides from *Harpadon nehereus* bone ameliorate sodium palmitate-induced HepG2 lipotoxicity by regulating oxidative stress and lipid metabolism. Mar Drugs. (2025) 23:3. doi: 10.3390/md23030118, 40137304 PMC11943913

[57] He RR Shi Y Lu XS Zhou YF Liu ZH Zhang S . Inhibitory effect and mechanism of epigallocatechin gallate on the differentiation of 3T3-L1 preadipocytes. Plant Foods Hum Nutr. (2024) 79:867–74. doi: 10.1007/s11130-024-01229-z, 39186142

[58] Higashikawa K Uehara R Horiguchi S Shibata Y Okubo N Mizuno Y . Thymidine phosphorylase imaging probe for differential diagnosis of metabolic dysfunction-associated steatohepatitis. Mol Imaging Biol. (2024) 26:1036–45. doi: 10.1007/s11307-024-01964-4, 39538096

[59] Jiang S Li HQ Zhang LWY Mu WP Zhang Y Chen TJ . Generic diagramming platform (GDP): a comprehensive database of high-quality biomedical graphics. Nucleic Acids Res. (2025) 53:D1670–6. doi: 10.1093/nar/gkae973, 39470721 PMC11701665

[60] Suren Garg S Kushwaha K Dubey R Gupta J. Association between obesity, inflammation and insulin resistance: insights into signaling pathways and therapeutic interventions. Diabetes Res Clin Pract. (2023) 200:110691. doi: 10.1016/j.diabres.2023.110691, 37150407

[61] Yudhani RD Sari Y Nugrahaningsih DAA Sholikhah EN Rochmanti M Purba AKR . In vitro insulin resistance model: a recent update. J Obes. (2023) 2023:1–13. doi: 10.1155/2023/1964732, 36714242 PMC9876677

[62] Baris A Eugenia FB Eubanks J Khou S Anand S. Thymidine phosphorylase facilitates retinoic acid inducible gene-I induced endothelial dysfunction. Cell Death Dis. (2023) 14:4. doi: 10.1038/s41419-023-05821-0 PMC10131517 37100811

[63] Cai B He ZL Liu DD Zhang YP Yin ZK Bao WJ . Thymidine phosphorylase participates in platelet activation and promotes inflammation in rheumatoid arthritis. Toxicol Appl Pharmacol. (2025) 495:117217. doi: 10.1016/j.taap.2024.117217 39732205

[64] Monroy Muñoz IE Angeles Martinez J Posadas Sánchez R Villarreal Molina T Alvarez León E Flores Dominguez C . PLA2G2A polymorphisms are associated with metabolic syndrome and type 2 diabetes mellitus. Results from the genetics of atherosclerotic disease Mexican study. Immunobiol. (2017) 222:10. doi: 10.1016/j.imbio.2016.08.014 27608594

[65] Hu M Chen Y Deng F Chang B Luo J Dong L . D-mannose regulates hepatocyte lipid metabolism via PI3K/Akt/mTOR signaling pathway and ameliorates hepatic steatosis in alcoholic liver disease. Front Immunol. (2022) 13:877650. doi: 10.3389/fimmu.2022.877650, 35464439 PMC9021718

[66] Wu H-Y Ji Z-H Xie W-Y Guo H-X Zheng Y Gao W . KLF4 promotes milk fat synthesis by regulating the PI3K-AKT-mTOR pathway and targeting FASN activation in bovine mammary epithelial cells. iScience. (2024) 27:109850. doi: 10.1016/j.isci.2024.109850, 38779481 PMC11108978

[67] Zhou X Hu X Zhang Z Lin S Lin X Zhou T . Xingxiao pill suppressed the progression of non-small cell lung cancer by targeting SREBP1/FASN–induced fatty acid biosynthesis via PI3K/AKT/mTOR signaling pathway. Cancer Manag Res. (2025) 17:24. doi: 10.2147/cmar.S510010 PMC12304450 40734684

