# Supplementary material for: S-HPABA, a marine fungal-derived bioactive compound, exhibits potent lipid-lowering efficacy and safety in modulating lipid dysregulation revealed by integrative lipidomics and molecular biology approaches
Source: Front Nutr. 2026 Oct 5;13:1855150. doi: 10.3389/fnut.2026.1855150 (PMC13677371; doi:10.3389/fnut.2026.1855150)
Supplement: Supplementary file 1 [file Table_1.DOCX]

Table S1 Primer sequences in RT-qPCR.

| Gene | Primers |  |
| --- | --- | --- |
| FASN | Forward | CTGTCAGCAAGTTTGTCGCC |
|  | Reverse | CACAGGGCTGACCAGGTTATT |
| TYMP | Forward | CCTCAAAGGAAGAGGCCCAG |
|  | Reverse | CCAGCATCGCCTGGAACTTA |
| PLA2G2A | Forward | CACGACTGCTGTTATGGGGA |
|  | Reverse | TTAAGCGAGTCACAGTCGGC |
| ALG2 | Forward | TTTCCACGCTCTCTGTGCTT |
|  | Reverse | CCAGTCAATTGGCCCTCGAT |
| SMPD3 | Forward | TTTCGAAGGGTCACCTCGTG |
|  | Reverse | GTGAAGAGCGAATGCTGCTG |
| GAPDH | Forward | AGAAACCTGCCAAGTATGATGAG |
|  | Reverse | TTGCTGTAACCGAACTCATTGTC |

Table S2 Coefficients of the OPLS-DA model in the positive and negative ion modes.

|  | Con VS Mod | | | | Mod VS S-HPABA | | | |
| --- | --- | --- | --- | --- | --- | --- | --- | --- |
|  | R^2^X | R^2^Y | Q^2^ | Q^2^ of 200 permutation tests | R^2^X | R^2^Y | Q^2^ | Q^2^ of 200 permutation tests |
| Positive ion mode | 0.913 | 1.000 | 0.999 | -0.465 | 0.727 | 0.999 | 0.993 | -0.475 |
| Negative ion mode | 0.879 | 0.998 | 0.995 | -0.533 | 0.881 | 0.999 | 0.997 | -0.861 |

Table S3 Details of differential metabolites.

| Num | Compound Name | RT | m/z | Calc. MW | Ion Mode | Error (ppm) | MSI Level | VIP | P | FC | Formular | Con/Mod | Mod/*S*-HPABA |
| --- | --- | --- | --- | --- | --- | --- | --- | --- | --- | --- | --- | --- | --- |
| 1 | Sphinganine | 0.687 | 302.30539 | 301.29813 | [M+H]^+^ | 0.17 | 2 | 1.0651 | 3.6078E-06 | 1.6867 | C_18_H_39_NO_2_ | down | up |
| 2 | PC(P-16:0/16:0) | 3.513 | 718.57495 | 717.56765 | [M+H]^+^ | 0.57 | 2 | 1.4231 | 1.0197E-06 | 1.3776 | C_40_H_80_NO_7_P | down | up |
| 3 | PC(20:5(5Z,8Z,11Z,14Z,17Z)/P-18:1(11Z)) | 2.322 | 790.57483 | 789.56755 | [M+H]^+^ | 0.39 | 2 | 1.1713 | 2.7508E-07 | 1.3021 | C_46_H_80_NO_7_P | down | up |
| 4 | Hypoxanthine | 0.565 | 137.04584 | 136.03858 | [M+H]^+^ | 0.54 | 2 | 3.1986 | 3.2834E-06 | 0.6932 | C_5_H_4_N_4_O | up | down |
| 5 | Palmitic acid | 0.644 | 274.27405 | 273.26676 | [M+H]^+^ | 0.52 | 2 | 1.4326 | 1.3401E-03 | 1.2673 | C_16_H_32_O_2_ | down | up |
| 6 | SM(d18:1/18:0) | 4.813 | 568.56641 | 567.55913 | [M+H]^+^ | 0.15 | 2 | 1.7732 | 5.0708E-04 | 6.2787 | C_36_H_73_NO_3_ | down | up |
| 7 | Oleoylethanolamide | 1.007 | 336.3262 | 353.32949 | [M+H-H_2_O]^+^ | 0.31 | 2 | 1.4653 | 2.8447E-09 | 1.3754 | C_22_H_43_NO_2_ | down | up |
| 8 | LysoPC(18:1/0:0) | 0.743 | 496.33975 | 495.33243 | [M+H]^+^ | -0.12 | 2 | 7.8285 | 7.7128E-13 | 1.7553 | C_24_H_50_NO_7_P | up | up |
| 9 | L-beta,gamma-Distearoyl-alpha-cephalin | 3.831 | 748.58521 | 747.57794 | [M+H]^+^ | 0.18 | 2 | 2.1596 | 4.9172E-09 | 1.4503 | C_41_H_82_NO_8_P | down | up |
| 10 | Eicosapentaenoyl PAF C-16 | 2.566 | 766.57446 | 765.56727 | [M+H]^+^ | 0.03 | 2 | 1.5507 | 2.1294E-10 | 1.2752 | C_44_H_80_NO_7_P | down | up |
| 11 | PE(16:0/16:0)-N,N-dimethyl | 3.153 | 720.55402 | 719.54672 | [M+H]^+^ | 0.3 | 2 | 2.0053 | 8.5582E-08 | 1.4643 | C_39_H_78_NO_8_P | down | up |
| 12 | PS(16:0/16:0) | 3.543 | 734.56982 | 733.56262 | [M+H]^+^ | 0.64 | 2 | 4.7870 | 4.5553E-10 | 1.2680 | C_40_H_80_NO_8_P | down | up |
| 13 | Cer(d18:1/18:0) | 4.791 | 566.55072 | 565.54342 | [M+H]^+^ | 0.04 | 2 | 1.6846 | 5.4909E-07 | 0.7890 | C_36_H_71_NO_3_ | up | down |
| 14 | 4-Oxo-4-(9H-purin-6-ylamino)butanoic acid | 0.56 | 236.07779 | 235.07051 | [M+H]^+^ | -0.12 | 2 | 1.4305 | 9.0871E-11 | 1.8672 | C_9_H_9_N_5_O_3_ | down | up |
| 15 | PG(16:0/18:2(9Z,12Z)) | 4.859 | 636.55621 | 618.52241 | [M+NH_4_]^+^ | 0.15 | 2 | 3.0270 | 1.2981E-11 | 0.6348 | C_39_H_70_O_5_ | up | down |
| 16 | 4-D-Hydroxysphinganine | 0.639 | 318.3002 | 317.29294 | [M+H]^+^ | -0.16 | 2 | 1.0199 | 1.6416E-02 | 1.2372 | C_18_H_39_NO_3_ | down | up |
| 17 | 2,3-Methyleneglutaric acid | 0.592 | 157.04956 | 156.04228 | [M+H]^+^ | 0.16 | 2 | 2.5893 | 1.7745E-05 | 1.3746 | C_7_H_8_O_4_ | down | up |
| 18 | DG(18:0/20:4(5Z,8Z,11Z,14Z)/0:0) | 5.261 | 662.57184 | 644.53809 | [M+NH_4_]^+^ | 0.18 | 2 | 2.4237 | 2.0563E-09 | 1.4275 | C_41_H_72_O_5_ | up | up |
| 19 | 1-Palmitoyl-2-linoleoyl PE | 3.027 | 716.52264 | 715.51539 | [M+H]^+^ | 0.26 | 2 | 1.8626 | 4.2515E-10 | 0.3960 | C_39_H_74_NO_8_P | up | down |
| 20 | DG(16:1(9Z)/18:1(9Z)/0:0) | 4.78 | 615.49597 | 592.50677 | [M+Na]^+^ | 0.16 | 3 | 1.1445 | 8.1582E-06 | 0.7524 | C_37_H_68_O_5_ | up | down |
| 21 | PC(18:1(9Z)/18:2(9Z,12Z)) | 3.403 | 784.58521 | 783.57794 | [M+H]^+^ | 0.17 | 2 | 2.6376 | 5.1653E-05 | 0.7123 | C_44_H_82_NO_8_P | up | down |
| 22 | PIP2(16:0/20:3(8Z,11Z,14Z)) | 4.124 | 812.61621 | 811.60909 | [M+H]^+^ | -0.02 | 2 | 1.9705 | 8.2149E-10 | 0.4631 | C_46_H_86_NO_8_P | up | down |
| 23 | PC(40:4) | 4.08 | 838.63202 | 837.62471 | [M+H]^+^ | -0.05 | 2 | 2.3382 | 1.1752E-11 | 0.4972 | C_48_H_88_NO_8_P | up | down |
| 24 | PC(18:0/22:5(4Z,7Z,10Z,13Z,16Z)) | 3.834 | 836.61633 | 835.60904 | [M+H]^+^ | -0.08 | 2 | 4.0940 | 6.4907E-12 | 0.6766 | C_48_H_86_NO_8_P | up | down |
| 25 | PC(40:6) | 3.127 | 834.60107 | 833.5938 | [M+H]^+^ | 0.41 | 2 | 3.1297 | 1.6699E-11 | 0.4461 | C_48_H_84_NO_8_P | up | down |
| 26 | PE(16:0/20:4(5Z,8Z,11Z,14Z)) | 2.785 | 740.52252 | 739.51527 | [M+H]^+^ | 0.08 | 2 | 3.3951 | 5.9246E-12 | 0.3638 | C_41_H_74_NO_8_P | up | down |
| 27 | PE(16:0/22:1(13Z)) | 3.892 | 774.60077 | 773.59365 | [M+H]^+^ | 0.25 | 2 | 2.3412 | 1.1923E-03 | 1.2769 | C_43_H_84_NO_8_P | down | up |
| 28 | LPC(17:0/0:0) | 0.789 | 510.35553 | 509.34825 | [M+H]^+^ | 0.21 | 2 | 1.7173 | 4.7796E-09 | 2.2321 | C_25_H_52_NO_7_P | down | up |
| 29 | 1-[(9Z)-octadecenoyl]-2-[(4Z,7Z,10Z,13Z,16Z,19Z)-docosahexaenoyl]-sn-glycero-3-phosphocholine | 2.486 | 832.58514 | 831.57778 | [M+H]^+^ | -0.03 | 2 | 5.5872 | 4.6837E-09 | 0.7710 | C_48_H_82_NO_8_P | up | down |
| 30 | 1-[(8Z,11Z,14Z)-icosatrienoyl]-sn-glycero-3-phosphocholine | 0.708 | 546.35553 | 545.34774 | [M+H]^+^ | -0.74 | 2 | 1.9342 | 1.9531E-12 | 0.2764 | C_28_H_52_NO_7_P | up | down |
| 31 | PE(18:4(6,9,12,15)/24:1(15)) | 2.453 | 822.60095 | 821.59358 | [M+H]^+^ | 0.15 | 2 | 1.2069 | 3.9218E-09 | 1.7744 | C_47_H_84_NO_8_P | down | up |
| 32 | PC(20:4/20:4) | 1.868 | 830.56934 | 829.56201 | [M+H]^+^ | -0.17 | 2 | 4.0986 | 5.1584E-15 | 0.4113 | C_48_H_80_NO_8_P | up | down |
| 33 | (9R)-6-Hydroxy-6-oxido-12-oxo-5,7,11-trioxa-2-aza-6lambda-5-phosphaheptacosan-9-yl palmitate | 2.659 | 706.53821 | 705.53105 | [M+H]^+^ | 0.27 | 2 | 1.4486 | 6.7772E-07 | 1.2953 | C_38_H_76_NO_8_P | down | up |
| 34 | (2R)-3-{[(2-Aminoethoxy)(hydroxy)phosphoryl]oxy}-2-[(9Z,12Z)-9,12-octadecadienoyloxy]propyl (7Z,10Z,13Z,16Z,19Z)-7,10,13,16,19-docosapentaenoate | 2.547 | 790.53839 | 789.531 | [M+H]^+^ | 0.19 | 2 | 4.0470 | 1.2243E-06 | 0.2668 | C_45_H_76_NO_8_P | up | down |
| 35 | (2R)-2-[(4Z,7Z,10Z,13Z,16Z,19Z)-4,7,10,13,16,19-Docosahexaenoyloxy]-3-[(4Z,7Z,10Z,13Z,16Z)-4,7,10,13,16-docosapentaenoyloxy]propyl 2-(trimethylammonio)ethyl phosphate | 1.921 | 880.58502 | 879.57783 | [M+H]^+^ | 0.03 | 2 | 1.8523 | 8.1919E-07 | 0.4103 | C_52_H_82_NO_8_P | up | down |
| 36 | (2R)-2,3-Bis[(9E)-9-octadecenoyloxy]propyl 2-(trimethylammonio)ethyl phosphate | 3.738 | 786.56439 | 785.59386 | [M+H]^+^ | 0.51 | 2 | 9.2150 | 1.0883E-02 | 0.7443 | C_44_H_84_NO_8_P | up | down |
| 37 | O-{[(2R)-2-[(7Z,10Z,13Z,16Z)-7,10,13,16-Docosatetraenoyloxy]-3-(heptadecanoyloxy)propoxy](hydroxy)phosphoryl}-L-serine | 5.325 | 824.54462 | 825.55187 | [M-H]^-^ | -0.14 | 2 | 1.1679 | 6.3792E-06 | 0.7681 | C_45_H_80_NO_10_P | down | down |
| 38 | O-(Hydroxy{(2R)-3-(pentadecanoyloxy)-2-[(15Z)-15-tetracosenoyloxy]propoxy}phosphoryl)-L-serine | 6.118 | 830.59161 | 831.59891 | [M-H]^-^ | -0.03 | 2 | 1.4972 | 5.5433E-05 | 0.6384 | C_45_H_86_NO_10_P | up | down |
| 39 | O-(Hydroxy{(2R)-2-hydroxy-3-[(2-methoxyicosyl)oxy]propoxy}phosphoryl)-L-serine | 1.529 | 568.36194 | 569.36929 | [M-H]^-^ | 0.04 | 2 | 2.8379 | 2.9799E-02 | 1.3383 | C_27_H_56_NO_9_P | up | up |
| 40 | O-(Hydroxy{(2R)-2-[(11Z,14Z)-11,14-icosadienoyloxy]-3-[(9Z)-9-nonadecenoyloxy]propoxy}phosphoryl)-L-serine | 5.397 | 826.56024 | 827.56756 | [M-H]^-^ | -0.09 | 2 | 1.0421 | 1.3547E-10 | 0.2805 | C_45_H_82_NO_10_P | up | down |
| 41 | Glucuronic acid-3,6-lactone | 0.539 | 175.02475 | 176.03202 | [M-H]^-^ | -0.37 | 2 | 1.1113 | 3.4913E-09 | 7.5612 | C_6_H_8_O_6_ | down | up |
| 42 | Eicosapentaenoic acid | 2.177 | 301.21719 | 302.22448 | [M-H]^-^ | -0.34 | 2 | 6.2412 | 1.8175E-04 | 1.4873 | C_20_H_30_O_2_ | down | up |
| 43 | Docosahexaenoic acid | 2.726 | 327.23291 | 328.24019 | [M-H]^-^ | -0.14 | 2 | 15.1928 | 1.8375E-05 | 2.1331 | C_22_H_32_O_2_ | down | up |
| 44 | Cholesterol sulfate | 3.792 | 465.30432 | 466.3116 | [M-H]^-^ | -0.16 | 2 | 4.8713 | 6.3387E-09 | 0.2940 | C_27_H_46_O_4_S | up | down |
| 45 | all-cis-4,7,10,13,16-Docosapentaenoic acid | 3.333 | 329.24857 | 330.25584 | [M-H]^-^ | -0.12 | 2 | 1.7741 | 2.1928E-02 | 1.7601 | C_22_H_34_O_2_ | up | up |
| 46 | PE(18:0/20:4(5Z,8Z,11Z,14Z)) | 6.266 | 766.53925 | 767.54652 | [M-H]^-^ | 0.03 | 2 | 2.6645 | 4.0489E-11 | 0.3675 | C_43_H_78_NO_8_P | up | down |
| 47 | PG(18:0/20:4) | 5.82 | 797.53412 | 798.54143 | [M-H]^-^ | 0.43 | 2 | 1.1367 | 1.2865E-10 | 0.4660 | C_44_H_79_O_10_P | down | down |
| 48 | PS(18:0/22:6(4Z,7Z,10Z,13Z,16Z,19Z)) | 5.584 | 834.52881 | 835.53609 | [M-H]^-^ | -0.29 | 2 | 1.7326 | 2.7232E-05 | 1.2837 | C_46_H_78_NO_10_P | down | up |
| 49 | LysoPE(18:2(9Z,12Z)/0:0) | 0.937 | 476.27823 | 477.28551 | [M-H]^-^ | -0.05 | 2 | 1.6134 | 3.2651E-15 | 0.1050 | C_23_H_44_NO_7_P | up | down |
| 50 | LPE(20:0) | 1.527 | 508.34085 | 509.34813 | [M-H]^-^ | -0.02 | 2 | 2.8513 | 2.9861E-02 | 1.3294 | C_25_H_52_NO_7_P | up | up |
| 51 | PE(16:0/22:6(4Z,7Z,10Z,13Z,16Z,19Z)) | 5.684 | 762.50781 | 763.5151 | [M-H]^-^ | -0.14 | 2 | 1.7969 | 4.1467E-08 | 0.6567 | C_43_H_74_NO_8_P | down | down |
| 52 | PE(P-16:1/22:6) | 5.91 | 746.51306 | 747.52035 | [M-H]^-^ | 0.08 | 2 | 1.6585 | 3.2276E-05 | 0.5032 | C_43_H_74_NO_7_P | down | down |
| 53 | LPC(17:1/0:0) | 1.14 | 506.3252 | 507.33247 | [M-H]^-^ | -0.04 | 2 | 4.5292 | 5.1537E-12 | 0.4777 | C_25_H_50_NO_7_P | up | down |
| 54 | 11,12-Epoxyeicosatrienoic acid | 1.249 | 319.22784 | 320.23512 | [M-H]^-^ | -0.09 | 2 | 1.1612 | 3.3923E-02 | 0.6206 | C_20_H_32_O_3_ | up | down |
| 55 | (2R)-3-{[(2-Aminoethoxy)(hydroxy)phosphoryl]oxy}-2-[(11Z)-11-octadecenoyloxy]propyl (9Z)-9-octadecenoate | 6.324 | 742.53943 | 743.54678 | [M-H]^-^ | 0.37 | 2 | 1.5032 | 5.6191E-12 | 0.3167 | C_41_H_78_NO_8_P | up | down |
| 56 | (2R)-3-({[(2S)-2,3-Dihydroxypropoxy](hydroxy)phosphoryl}oxy)-2-[(4Z,7Z,10Z,13Z,16Z,19Z)-4,7,10,13,16,19-docosahexaenoyloxy]propyl (4Z,7Z,10Z,13Z,16Z,19Z)-4,7,10,13,16,19-docosahexaenoate | 4.532 | 865.5025 | 866.50975 | [M-H]^-^ | -0.04 | 2 | 1.4187 | 3.3148E-07 | 1.5213 | C_50_H_75_O_10_P | down | up |
| 57 | (2R)-1-{[(2-Aminoethoxy)(hydroxy)phosphoryl]oxy}-3-hydroxy-2-propanyl (6Z,9Z,12Z,15Z,18Z,21Z)-6,9,12,15,18,21-tetracosahexaenoate | 0.869 | 552.30975 | 553.31692 | [M-H]^-^ | 0.14 | 2 | 2.9202 | 1.9664E-12 | 0.3602 | C_29_H_48_NO_7_P | down | down |

Table S4 Details of the metabolites and pathways associated with the key genes.

| Gene | Compound Name | Pathway |
| --- | --- | --- |
| PLA2G2A | PC(P-16:0/16:0) | Glycerophospholipid metabolism |
|  | PC(20:5(5Z,8Z,11Z,14Z,17Z)/P-18:1(11Z)) | Arachidonic acid metabolism |
|  | PC(18:1(9Z)/18:2(9Z,12Z)) | Linoleic acid metabolism |
|  | PC(40:4) | α-linolenic acid metabolism |
|  | PC(18:0/22:5(4Z,7Z,10Z,13Z,16Z)) | GPI anchor biosynthesis |
|  | PC(40:6) |  |
|  | PC(20:4/20:4) |  |
|  | PE(16:0/16:0)-N,N-dimethyl |  |
|  | PE(16:0/20:4(5Z,8Z,11Z,14Z)) |  |
|  | PE(16:0/22:1(13Z)) |  |
|  | PE(18:4(6,9,12,15)/24:1(15)) |  |
|  | PE(18:0/20:4(5Z,8Z,11Z,14Z)) |  |
|  | PE(16:0/22:6(4Z,7Z,10Z,13Z,16Z,19Z)) |  |
|  | PE(P-16:1/22:6) |  |
|  | PS(16:0/16:0) |  |
|  | PS(18:0/22:6(4Z,7Z,10Z,13Z,16Z,19Z)) |  |
|  | LysoPC(18:1/0:0) |  |
| FASN | Palmitic acid | Biosynthesis of unsaturated fatty acids |
|  |  | GPI anchor biosynthesis |
|  |  | Fatty acid degradation |
|  |  | Fatty acid biosynthesis |
| TYMP | Hypoxanthine | Purine metabolism |
| SMPD3 | Cer(d18:1/18:0) | Sphingolipid metabolism |
|  | SM(d18:1/18:0) |  |
| ALG2 | PE(16:0/16:0)-N,N-dimethyl | Glycerophospholipid metabolism |
|  | PE(16:0/20:4(5Z,8Z,11Z,14Z)) | GPI anchor biosynthesis |
|  | PE(16:0/22:1(13Z)) |  |
|  | PE(18:4(6,9,12,15)/24:1(15)) |  |
|  | PE(18:0/20:4(5Z,8Z,11Z,14Z)) |  |
|  | PE(16:0/22:6(4Z,7Z,10Z,13Z,16Z,19Z)) |  |
|  | PE(P-16:1/22:6) |  |

Fig. S1 The chemical structure and synthesis route of *S*-HPABA.

Fig. S2 TIC diagram of QC samples in positive and negative ion modes.

Fig. S3 Fitting performance of the OPLS-DA model. **A-B** 200 replacement tests in positive ion mode. **C-D** 200 replacement tests under negative ion conditions.

Fig. S4 Screening of differential metabolites. **A-B, E-F** VIP diagram in positive and negative ion conditions. **C-D, G-H** Volcano plot under positive and negative ion parameters.

Fig. S5 Structural comparison between *S*-HPABA and aspirin.
